# Rad51/Dmc1 paralogs and mediators oppose DNA helicases to limit hybrid DNA formation and promote crossovers during meiotic recombination

**DOI:** 10.1093/nar/gku1219

**Published:** 2014-11-20

**Authors:** Alexander Lorenz, Alizée Mehats, Fekret Osman, Matthew C. Whitby

**Affiliations:** 1 Department of Biochemistry, University of Oxford, South Parks Road, Oxford OX1 3QU, UK; 2 The Institute of Medical Sciences (IMS), University of Aberdeen, Foresterhill, Aberdeen AB25 2ZD, UK

## Abstract

During meiosis programmed DNA double-strand breaks (DSBs) are repaired by homologous recombination using the sister chromatid or the homologous chromosome (homolog) as a template. This repair results in crossover (CO) and non-crossover (NCO) recombinants. Only CO formation between homologs provides the physical linkages guiding correct chromosome segregation, which are essential to produce healthy gametes. The factors that determine the CO/NCO decision are still poorly understood. Using *Schizosaccharomyces pombe* as a model we show that the Rad51/Dmc1-paralog complexes Rad55-Rad57 and Rdl1-Rlp1-Sws1 together with Swi5-Sfr1 play a major role in antagonizing both the FANCM-family DNA helicase/translocase Fml1 and the RecQ-type DNA helicase Rqh1 to limit hybrid DNA formation and promote Mus81-Eme1-dependent COs. A common attribute of these protein complexes is an ability to stabilize the Rad51/Dmc1 nucleoprotein filament, and we propose that it is this property that imposes constraints on which enzymes gain access to the recombination intermediate, thereby controlling the manner in which it is processed and resolved.

## INTRODUCTION

In order to enable correct chromosome segregation during meiosis, the homologous chromosomes have to pair up and get physically linked. This is achieved via the repair of programmed DNA double-strand breaks (DSBs) by homologous recombination. Specifically, repair has to occur using the homolog rather than the sister chromatid as a template (interhomolog recombination) and critically at least some of the interhomolog recombination intermediates have to be processed into crossing overs (1,2).

The crossover (CO)/non-crossover (NCO) decision is a key point for regulation of meiotic recombination, and one that is inextricably linked to the strand exchange reaction driven by Rad51 and/or Dmc1. These proteins load onto single-stranded (ss) DNA, which is formed by nucleolytic resection of the DSB (3). Multiple protomers are loaded creating proteinaceous filaments that encase the DNA. Within these nucleoprotein filaments intact homologous DNA partners are located, paired with the broken DNA and then invaded. Strand invasion forms a displacement (D) loop at which DNA synthesis is primed leading to extension of the invading strand (4,5). The recombination reaction can then take one of the several different paths: the D-loop can be unwound allowing the extended DNA strand to anneal to its complementary strand at the other end of the break in a process known as synthesis-dependent strand annealing (SDSA); the D-loop can be cleaved; or the ssDNA tail at the other end of the DSB can anneal to the displaced strand of the D-loop in a process termed second end capture. SDSA results in an NCO, whereas D-loop cleavage gives rise to a CO. Second end capture leads to the formation of a double Holliday junction (HJ) (or in fission yeast a single HJ seemingly due to nicking of the displaced strand prior to second end capture), which can branch migrate extending the amount of hybrid DNA that is formed (6–9). HJs are resolved by structure-specific endonucleases generating either COs or NCOs depending on which DNA strands are cleaved (10,11). Key factors involved in these processes include the DNA helicase/translocase FANCM (Fml1 in *Schizosaccharomyces pombe*), RecQ-type helicases and the Mus81-Eme1 endonuclease (12–20).

*In vitro* FANCM can unwind D-loops and branch migrate HJs, and in plants and fission yeast appears to utilize these activities during meiosis to direct repair via SDSA with the help of its histone-fold co-factors MHF1 and MHF2 (16–18,21–24). RecQ-type helicases, which can also branch migrate HJs, perform a wide range of regulatory roles in homologous recombination including the promotion of NCO formation during meiosis, at least in *Saccharomyces cerevisiae* and *Tetrahymena thermophila* (19,20,25). Mus81-Eme1 can cleave a variety of branched DNA molecules *in vitro*, including D-loops, nicked HJs and fully ligated HJs, albeit its ability to cleave the latter junction remains contentious (12,26–30). *In vivo* it can promote both CO and NCO formation, and at least in fission yeast is thought to bias resolution toward COs by cleaving D-loops (12–14,16,20,28,31–34). The factors that govern which of these enzymes are used to resolve meiotic recombination intermediates remain largely unknown.

In fission yeast we recently identified the Swi5–Sfr1 complex as a determinant of the CO/NCO decision, seemingly functioning to counteract Fml1-Mhf1-Mhf2 and thereby assigning recombination intermediates to be processed by Mus81-Eme1 (7,12,16). Swi5–Sfr1 mediates Dmc1 loading on to RPA (replication protein A)-coated ssDNA and enhances the stability of both Rad51- and Dmc1-nucleoprotein filaments (35). This latter activity in particular could control the accessibility of the D-loop to Fml1, Mus81-Eme1 and potentially other proteins. However, Swi5–Sfr1 is not the only protein complex that mediates the formation of the Rad51/Dmc1-nucleoprotein filament or enhances its stability. In budding yeast the Rad51-paralog complexes, Rad55-Rad57 and Psy3-Csm2, perform similar functions (36,37).

In this study we employ genetic assays to determine whether the Rad51/Dmc1 paralogs and mediators are determinants of the CO/NCO decision in fission yeast. We find that, along with Swi5-Sfr1, both Rad55-Rad57 and Rdl1-Rlp1 (the fission yeast homologs of Psy3-Csm2) together with Sws1 promote CO formation by antagonizing both Fml1 and the RecQ-type helicase Rqh1. Based on these findings we propose that Rad51/Dmc1 nucleoprotein filament stability is a major determinant of the CO/NCO decision by imposing constraints on which junction processing enzymes can gain access to the underlying DNA.

## MATERIALS AND METHODS

### Yeast strains and plasmid construction

*S. pombe* strains used for this study are listed in Supplementary Table S1. Yeast cells were cultured on yeast extract (YE), on pombe minimal glutamate (PMG) and on yeast nitrogen base glutamate (YNG) agar plates containing the required supplements (concentration 250 μg/ml on YE, 75 μg/ml on PMG and YNG). Crosses were performed on malt extract (ME) agar with the required amino acids (concentration 50 μg/ml). Determination of spore viability by random spore analysis and the meiotic recombination assay have been previously described in detail (12,16,38,39).

To make a *sfr1* deletion strain marked with a hygromycin B resistance, the *hphMX4* cassette from pAG32 (40) was subcloned as a SacI-BamHI fragment into a plasmid carrying up- and downstream flanking sequences of the *sfr1* open reading frame (ORF) (16). The deletion cassette was released from the plasmid by digesting with HindIII and transformed into FO652 using a standard Li-acetate protocol (41).

The original *sws1*Δ strain VM3723 is *his3*^+^ (42). Due to the proximity of the *his3* and *sws1* genes (they are only separated by ∼5 kb on chromosome II) it was unfeasible to create a *sws1*Δ::*kanMX6 his3-D1* strain by crossing. Using oligos located several 100 bp up- and downstream of *sws1* (oMW1540 5′-GATCAAATAACTCTCGAAGC-3′ and oMW1541 5′-CTTGATCAAACCTAGACG-3′) a *sws1*-deletion cassette was PCR-amplified using Phusion High-Fidelity DNA polymerase (Thermo Fisher Scientific Inc., Waltham, MA, USA) and genomic DNA from VM3723 as a template. The polymerase chain reaction (PCR) product was then transformed into MCW1221 using a standard Li-acetate protocol (41).

To produce *rdl1*Δ and *rlp1*Δ strains marked with a nourseothricin resistance and a *sws1*Δ strain marked with a hygromycin B resistance we used a one-step marker swap protocol. This makes use of the homology provided by the *TEF*-promoter and -terminator sequences present in all the *MX* constructs. Cassettes released by endonuclease digest from plasmids pCR2.1-nat (EcoRI) and pAG32 (BamHI-EcoRV) were used to swap markers in *rdl1*Δ::*kanMX6*, *rlp1*Δ::*kanMX6* and *sws1*Δ::*kanMX6* strains (40,43).

All restriction endonucleases were obtained from New England BioLabs (NEB), Inc. (Ipswich, MA, USA).

### Construction of a meiotic recombination reporter system flanking *ade7*

Similar to the meiotic recombination assay at *ade6*, where we utilized different point mutations (both hotspots and non-hotspots) (12,16,44), we integrated *his3*^+^ and *ura4*^+^ markers up- and downstream of *ade7*. The point mutations in *ade7-50* and *ade7-152* were verified by DNA sequencing, and a discrepancy to the previously reported sequence for *ade7-50* was noted; T779del/G780C rather than G780C (45).

DNA sequences ∼6.8 kb upstream (oMW1511 5′-aattaaggatccaacctcattcctctccc-3′ and oMW1512 5′-aattaaactagtggggacgacgagtcg-3′) and ∼12.4 kb downstream (oMW1513 5′-aattaaggatcctcaatttcttgcagttcc-3′ and oMW1514 5′- aattaaactagtctcaagcctcaaacaacc-3′) of the *ade7* ORF were PCR-amplified from ALP1596 using Phusion High-Fidelity DNA polymerase, cut with SpeI, and cloned into pAG25 (40) cut with SpeI and PvuII. The *his3*^+^-marker was PCR-amplified from pFOX2 (46) (oMW1515 5′-gcttggctgcaggaattc-3′ and oMW1516 5′- aattaactgcagtatcgataagcttgatggc-3′) creating a *his3* ORF flanked by two PstI sites, this construct was then cloned into the PstI site of the plasmid carrying the *ade7*-upstream flanking fragment (giving pALo109). The *ura4*^+^-marker was subcloned as a HindIII-fragment from pDUP12 (47) into the HindIII site of the plasmid carrying the *ade7*-downstream flanking region (giving pALo111). All relevant plasmid sections were verified by DNA sequencing. The cassettes for transformation were released from the plasmids by restriction digest. The ∼3.2-kb EagI-SpeI fragment from pALo109 and the ∼2.5-kb BamHI-SpeI fragment from pALo111 were then introduced into ALP1596 and ALP1593, respectively, by a standard Li-acetate transformation protocol (41).

All restriction endonucleases were obtained from New England BioLabs (NEB), Inc. (Ipswich, MA).

### Data presentation and statistics

Data presented as box-and-whisker plots were created in R (version i386, 3.0.1) (http://www.r-project.org/) using the boxplot() function with its standard settings. The lower and upper ‘hinges’ of the box represent the first and the third quartile, respectively, and the black bar within the box indicates the median ( = second quartile). The ‘whiskers’ represent the minimum and maximum of the range, unless they differ more than 1.5 times the interquartile distance from the median. In the latter case, the borders of the 1.5 times interquartile distance around the median are indicated by the ‘whiskers’ and values outside this range (‘outliers’) are shown as open circles. Raw data and R scripts used for the boxplot() function are available online as supporting material (48).

Several data sets did not conform to a normal distribution according to a Shapiro–Wilk test (http://sdittami.altervista.org/shapirotest/ShapiroTest.html). Therefore, all comparisons were done using a two-tailed Mann–Whitney U test, which is non-parametric and does not depend on data sets being normally distributed. Statistical analysis of the genetic recombination data was performed in G*Power 3.1.7 (49,50). *P* values were calculated with a given statistical power of 1 – β = 0.8.

## RESULTS

### Assay for monitoring meiotic recombination

**Figure 1.**
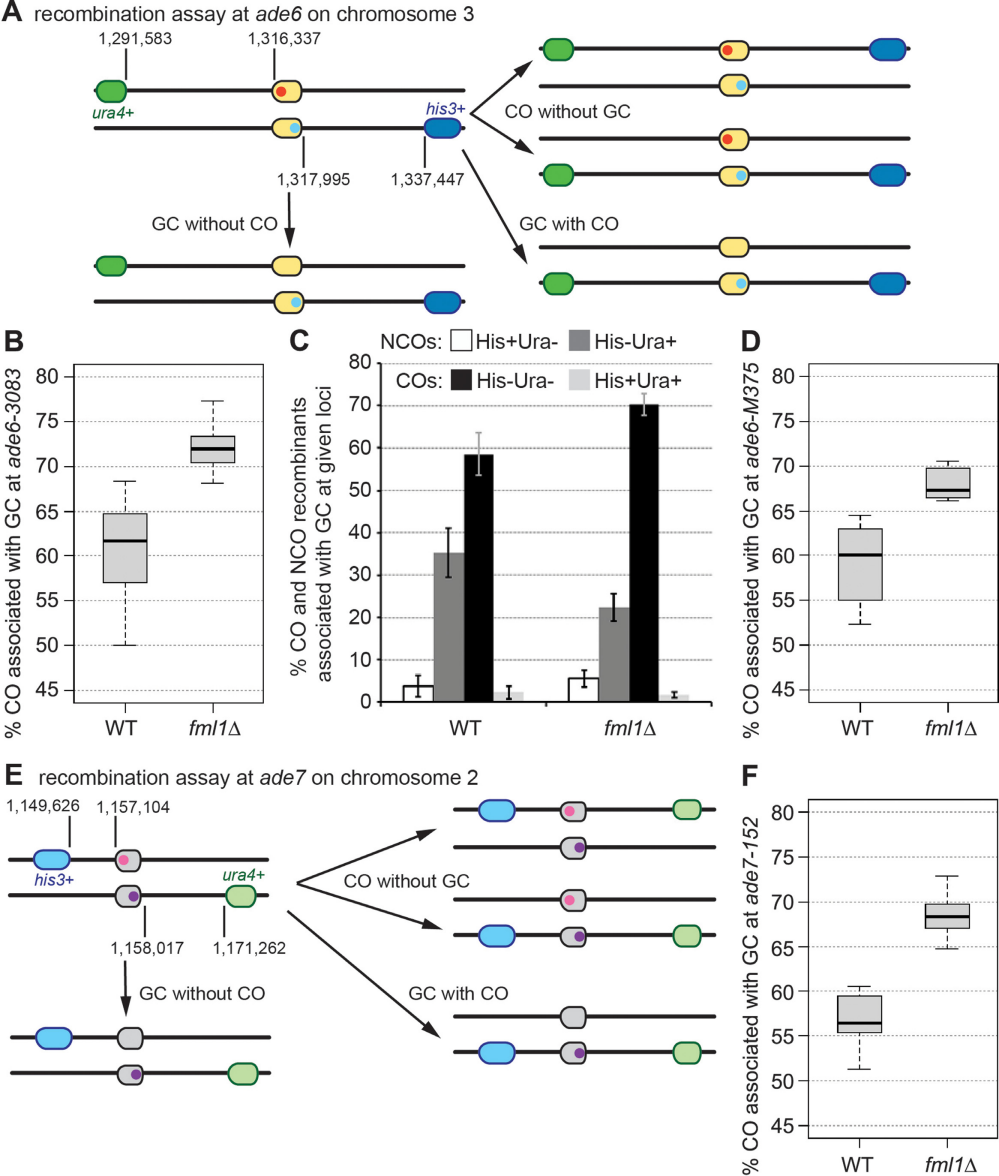
Fml1 is necessary for wild-type levels of non-crossovers associated with gene conversion events at recombination hotspots and non-hotspots. (**A**) Schematic showing the meiotic recombination assay at *ade6* (yellow) and its common outcomes. The positions of *ade6* and the artificially introduced markers *his3*^+^-*aim* (blue) and *ura4*^+^-*aim2* (green) on chromosome 3 are indicated (in bp). Point mutations *ade6-M375* and *-3083* are shown in red, and *ade6-469* is labeled in light blue. (**B**) Frequency of crossovers and (**C**) frequencies of different crossover and non-crossover classes associated with a gene conversion event in wild type and *fml1*Δ at *ade6-3083*×*ade6-469*; ALP733×ALP731 (WT, *n* = 41), ALP1133×FO2608 (*fml1*Δ, *n* = 15). (**D**) Frequency of crossovers associated with a gene conversion event in wild type and *fml1*Δ at *ade6-M375*×*ade6-469*; ALP1541×ALP731 (WT, *n* = 6), ALP1542×FO2608 (*fml1*Δ, *n* = 6). (**E**) Schematic of the meiotic recombination assay at *ade7* (gray) and its common outcomes. The location of *ade7* and the artificially introduced markers *his3*^+^-*aim3* (teal) and *ura4*^+^-*aim6* (light green) on chromosome 2 are shown (in bp). Point mutations *ade7-152* and *-50* are labeled in pink and purple, respectively. (**F**) Frequency of crossovers associated with a gene conversion event in wild type and *fml1*Δ at *ade7-152*×*ade7-50*; ALP1638×ALP1636 (WT, *n* = 12), ALP1670×ALP1669 (*fml1*Δ, *n* = 12). *n* indicates the number of independent crosses (see also Supplementary Table S2).

We have previously described a genetic recombination assay featuring intragenic markers (*ade6-3083* and *ade6-469*) and flanking intergenic markers (*his3^+^-aim* and *ura4^+^-aim2*) to identify and characterize determinants of template choice and the CO/NCO decision during meiotic recombination (12,16,44) (Figure 1A). Reductions in gene conversion (GC) and CO frequencies observed in this assay indicate either a lower frequency of DSB formation at the *ade6-3083* hotspot allele or reduced interhomolog recombination, whereas the genetic readout for the CO/NCO decision is the percentage of COs associated with a GC event (CO-GC). The linkage of the flanking intergenic markers to the GC event can also provide information about the site of recombination intermediate resolution relative to the position of the intragenic markers (Supplementary Figure S1). In wild-type crosses ∼60% of all *ade6^+^* GC events are also a CO between the flanking *his3^+^* and *ura4^+^* markers, and almost all of these COs are His^−^ Ura^−^ (Figure 1B and C). The remaining *ade6^+^* NCO recombinants are mostly His^−^ Ura^+^ (Figure 1B and C). This bias toward COs can be explained by the resolution of D-loops and/or unligated HJs by Mus81-Eme1 (Supplementary Figure S1—scenarios 2 and 7), whereas the NCOs likely stem from a combination of SDSA driven by Fml1 (Supplementary Figure S1—scenario 1) and HJ resolution by Mus81-Eme1, which gives equal numbers of COs and NCOs (Supplementary Figure S1—scenarios 3 and 8, and 4 and 9) (12,16,28,44).

The contribution of Fml1 in directing recombination intermediates toward an NCO outcome is indicated by the increase in CO-GCs in *fml1*Δ crosses (Figure 1B and C), which is observed with both the *ade6-3083* hotspot allele and the *ade6-M375* non-hotspot allele indicating that it is independent of the level of GC (compare Figure 1B and D; Supplementary Table S2) (16). To corroborate this at an independent locus on a different chromosome, we constructed an equivalent recombination assay at *ade7* (Figure 1E). Similar to what is seen at *ade6* CO formation associated with GCs at *ade7* is significantly increased in the absence of *fml1* (compare Figure 1B, D and F).

### Destabilization of the strand exchange process allows Mus81-independent repair of meiotic DSBs

**Figure 2.**
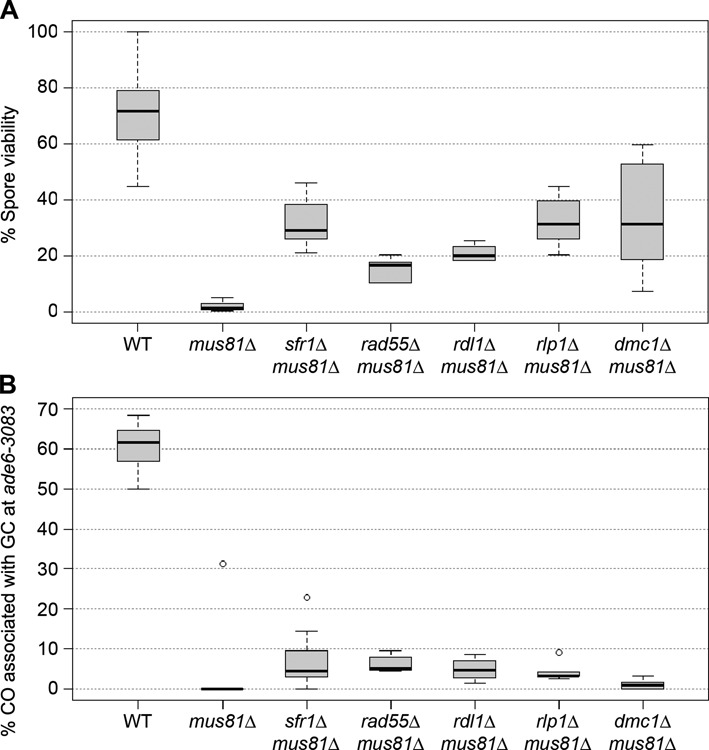
Deletion of strand exchange mediator genes and *dmc1* rescue the poor spore viability of a *mus81*Δ without the formation of crossovers. (**A**) Viability of progeny from wild-type and mutant crosses; ALP733×ALP731 (WT, *n* = 20), ALP812×ALP813 (*mus81*Δ, *n* = 10), ALP820×ALP814 (*sfr1*Δ *mus81*Δ, *n* = 10), ALP1674×ALP1673 (*rad55*Δ *mus81*Δ, *n* = 6), ALP1676×ALP1675 (*rdl1*Δ *mus81*Δ, *n* = 4), ALP1680×ALP1679 (*rlp1*Δ *mus81*Δ, *n* = 6), ALP1672×ALP1671 (*dmc1*Δ *mus81*Δ, *n* = 12). (**B**) Frequency of crossovers associated with a gene conversion event in wild-type and mutant crosses (*ade6-3083*×*ade6-469*); ALP733×ALP731 (WT, *n* = 41), ALP802×ALP822 (*mus81*Δ, *n* = 10), ALP824×ALP823 (*sfr1*Δ *mus81*Δ, *n* = 19), ALP1674×ALP1673 (*rad55*Δ *mus81*Δ, *n* = 6), ALP1676×ALP1675 (*rdl1*Δ *mus81*Δ, *n* = 4), ALP1680×ALP1679 (*rlp1*Δ *mus81*Δ, *n* = 6), ALP1672×ALP1671 (*dmc1*Δ *mus81*Δ, *n* = 12). *n* indicates the number of independent crosses (see also Supplementary Table S3).

In fission yeast Mus81-Eme1 is the structure-selective endonuclease solely responsible for meiotic CO formation. In line with this essential role the deletion of *mus81* causes meiotic catastrophe, which results in a dramatic reduction in spore viability, and an almost complete lack of CO recombinants among the few survivors (Figure 2) (12,51). Deletion of the Rad51/Dmc1 mediators Swi5-Sfr1 and Rad55-Rad57 in a *mus81*Δ background partially rescues spore viability (Figure 2A) (16,52,53). However, the formation of CO-GCs is not restored in *sfr1*Δ *mus81*Δ double mutants (*P* = 0.689) (Supplementary Table S3) (16). We have shown that in this situation Fml1 is capable of processing recombination intermediates into NCOs indicating that Swi5-Sfr1 normally blocks Fml1 from acting on joint molecules thereby biasing the recombination outcome toward CO formation by Mus81-Eme1 processing (16). This ability to block NCO formation is seemingly not restricted to Swi5-Sfr1 as deleting any of the Rad51 paralogs, including Dmc1, also rescues the poor spore viability of a *mus81* mutant to a similar degree and without increasing CO formation significantly (Figure 2 and Supplementary Table S3). This suggests that any destabilization of the strand exchange process alleviates the block against DNA helicases processing recombination intermediates into NCOs.

### Rad51 paralogs and Swi5-Sfr1 promote full activation of meiotic recombination independent of each other

**Figure 3.**
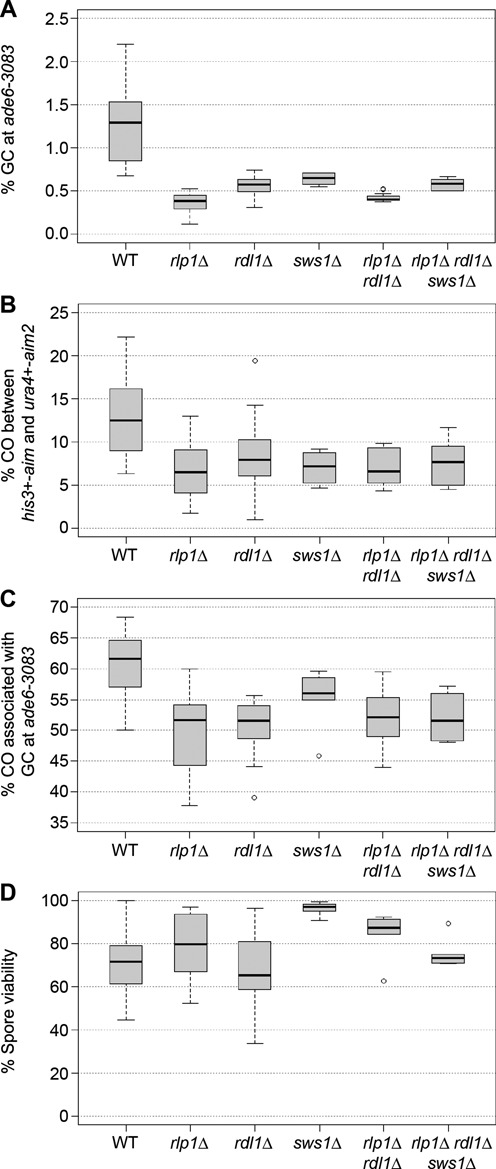
*rlp1*, *rdl1* and *sws1* are epistatic for meiotic recombination. (**A**) Percentage of gene conversions in wild-type and mutant crosses (*ade6-3083*×*ade6-469*); ALP733×ALP731 (WT, *n* = 41), ALP1623×ALP1620 (*rlp1*Δ, *n* = 18), ALP1621×ALP1611 (*rdl1*Δ, *n* = 18), ALP1708×ALP1707 (*sws1*Δ, *n* = 6), ALP1690×ALP1689 (*rlp1*Δ *rdl1*Δ, *n* = 12), ALP1733×ALP1732 (*rlp1*Δ *rdl1*Δ *sws1*Δ, *n* = 6). (**B**) Frequency of crossovers between *his3*^+^-*aim* and *ura4*^+^-*aim2* in wild-type and mutant meioses; crosses as in (A). (**C**) Frequency of crossovers associated with a gene conversion event in wild-type and mutant crosses (*ade6-3083*×*ade6-469*); crosses as in (A). (**D**) Viability of progeny from wild-type and mutant crosses; ALP733×ALP731 (WT, *n* = 20), ALP1623×ALP1620 (*rlp1*Δ, *n* = 17), ALP1621×ALP1611 (*rdl1*Δ, *n* = 18), ALP1708×ALP1707 (*sws1*Δ, *n* = 5), ALP1690×ALP1689 (*rlp1*Δ *rdl1*Δ, *n* = 6), ALP1733×ALP1732 (*rlp1*Δ *rdl1*Δ *sws1*Δ, *n* = 6). *n* indicates the number of independent crosses (see also Supplementary Table S4).

In order to assess the contribution of each Rad51 paralog or mediator to meiotic recombination, and determine whether there are any overlaps in function, we performed epistasis analyses. First we measured meiotic recombinant frequencies in *rlp1*Δ, *rdl1*Δ and *sws1*Δ single mutants and compared these to those of a *rlp1*Δ *rdl1*Δ double mutant and *rlp1*Δ *rdl1*Δ *sws1*Δ triple mutant (Figure 3A–C). In each case similar modest reductions in GC (2.0- to 3.6-fold), CO (1.6- to 2.1-fold) and CO-GC percentage were observed compared to wild type, which were not associated with any decline in spore viability (Figure 3D). All these differences in recombination frequencies were statistically significant, except the CO-GC of *sws1*Δ (Supplementary Table S4). These data are consistent with Rlp1, Rdl1 and Sws1 functioning as a complex to promote meiotic recombination similar to their homologs in budding yeast (37,54).

**Figure 4.**
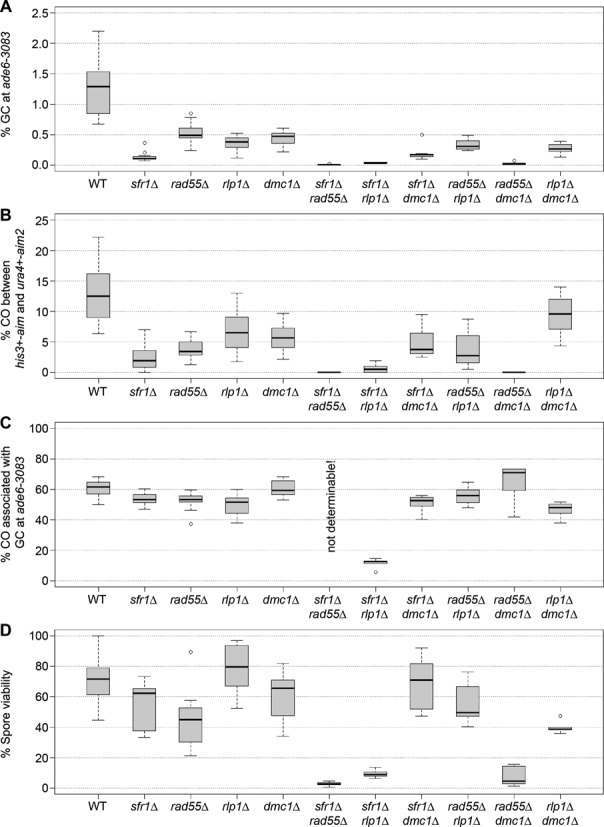
Strand exchange mediators are needed for wild-type levels of meiotic recombination. (**A**) Percentage of gene conversions in wild-type and mutant crosses (*ade6-3083*×*ade6-469*); ALP733×ALP731 (WT, *n* = 41), ALP800×ALP782 (*sfr1*Δ, *n* = 22), ALP1649×ALP1648 (*rad55*Δ, *n* = 12), ALP1623×ALP1620 (*rlp1*Δ, *n* = 18), ALP1545×ALP1544 (*dmc1*Δ, *n* = 12), ALP1735×ALP1734 (*sfr1*Δ *rad55*Δ, *n* = 6), ALP1700×ALP1699 (*sfr1*Δ *rlp1*Δ, *n* = 5), ALP1588×ALP1587 (*sfr1*Δ *dmc1*Δ, *n* = 12), ALP1704×ALP1703 (*rad55*Δ *rlp1*Δ, *n* = 12), ALP1696×ALP1695 (*rad55*Δ *dmc1*Δ, *n* = 6), ALP1694×ALP1693 (*rlp1*Δ *dmc1*Δ, *n* = 12). (**B**) Frequency of crossovers between *his3*^+^-*aim* and *ura4*^+^-*aim2* in wild-type and mutant meioses; crosses as in (A). (**C**) Frequency of crossovers associated with a gene conversion event in wild-type and mutant crosses (*ade6-3083*×*ade6-469*); crosses as in (A). (**D**) Viability of progeny from wild-type and mutant crosses; ALP733×ALP731 (WT, *n* = 20), ALP800×ALP782 (*sfr1*Δ, *n* = 10), ALP1649×ALP1648 (*rad55*Δ, *n* = 11), ALP1623×ALP1620 (*rlp1*Δ, *n* = 17), ALP1545×ALP1544 (*dmc1*Δ, *n* = 10), ALP1735×ALP1734 (*sfr1*Δ *rad55*Δ, *n* = 6), ALP1700×ALP1699 (*sfr1*Δ *rlp1*Δ, *n* = 5), ALP1588×ALP1587 (*sfr1*Δ *dmc1*Δ, *n* = 12), ALP1704×ALP1703 (*rad55*Δ *rlp1*Δ, *n* = 12), ALP1696×ALP1695 (*rad55*Δ *dmc1*Δ, *n* = 6), ALP1694×ALP1693 (*rlp1*Δ *dmc1*Δ, *n* = 5). *n* indicates the number of independent crosses (see also Supplementary Table S5).

Next, we compared *sfr1*Δ, *rad55*Δ and *rlp1*Δ single mutants (Figure 4). As previously noted *sfr1*Δ exhibits a strong reduction in GC (10-fold, *P* < 3.3 × 10^−16^) and overall CO (6-fold, *P* = 3.84 × 10^−13^) frequency, which has been attributed to a reduction in interhomolog recombination (53). In comparison, deleting *rad55* has only a moderate, but highly significant, effect on the general meiotic recombination rate similar to *rlp1*Δ (Figure 4A–C and Supplementary Table S5). None of these decreases in recombination frequency causes major problems in spore viability, indicating that DSBs are repaired with reasonable efficiency, and each of the three homolog pairs receives sufficient COs to support proper chromosome segregation (Figure 4D). Analysis of the double mutant combinations reveals an epistatic relationship between *rad55*Δ and *rlp1*Δ, while both *sfr1*Δ *rad55*Δ and *sfr1*Δ *rlp1*Δ double mutants exhibit synergistic reductions in GC (in excess of 39-fold compared to wild type), CO (more than 22-fold compared to wild type) and spore viability (at least 7.5-fold lower than in wild type) (Figure 4 and Supplementary Table S5). The *sfr1*Δ *rlp1*Δ double mutant also displays a large (5.4-fold) decrease in CO-GC (Figure 4C and Supplementary Table S5). Due to low mating efficiency and strongly reduced spore viability only very few Ade^+^ recombinants could be recovered from crosses of *sfr1*Δ *rad55*Δ double mutants, which makes it unfeasible to determine the CO-GC rate. Together these data indicate that Rad55-Rad57 and Swi5-Sfr1 heterodimers can promote CO-GC events independently of each other, suggesting separate contributions to CO-GC recombination events in wild-type cells. This is consistent with previous findings (52,53,55). The Rlp1–Rdl1–Sws1 complex appears to function together with Rad55-Rad57, which suggests that they contribute to a shared function.

To further define the relationship between these factors, we analyzed their genetic interaction with *dmc1* (Figure 4). Loss of *dmc1* causes a modest ∼2-fold reduction in GC (*P* = 2.88 × 10^−8^) and CO (*P* = 1.14 × 10^−5^), while retaining a wild-type level of spore viability (Figure 4A, B and D and Supplementary Table S5), however, unlike *sfr1*Δ, *rad55*Δ and *rlp1*Δ, the CO-GC percentage is not reduced (*P* = 0.795) (Figure 4C and Supplementary Table S5). The combination of *sfr1*Δ and *dmc1*Δ mutations results in an epistatic interaction displaying a *sfr1*Δ phenotype (Figure 4A and B). It has previously been noted that the levels of interhomolog recombination intermediate in a *dmc1*Δ *swi5*Δ double mutant lie between those of *dmc1*Δ and *swi5*Δ single mutants (52). Accordingly we observe a slight increase of GC and CO percentage in the *dmc1*Δ *sfr1*Δ double mutant compared with the *sfr1*Δ single mutant, however, the change in GC frequency is not statistically significant (*P* = 0.354), and the decrease in overall CO percentage is only weakly so (*P* = 0.065). In contrast a *rad55*Δ *dmc1*Δ double mutant exhibits similar low levels of GC, CO and spore viability as a *sfr1*Δ *rad55*Δ double mutant. These data indicate that Swi5-Sfr1 and Dmc1 function together to promote meiotic recombination, while Rad55-Rad57 contributes by distinct means.

Intriguingly, *rlp1*Δ and *rdl1*Δ exhibit a quite different genetic interaction with *dmc1*Δ than either *sfr1*Δ or *rad55*Δ, with epistasis for GC and CO frequency but a slightly reduced percentage of CO-GCs compared to either single mutant (*P* = 0.487 for *dmc1*Δ *rlp1*Δ versus *rlp1*Δ, and *P* = 0.045 for *dmc1*Δ *rdl1*Δ versus *rdl1*Δ) (Figure 4A–C , Supplementary Figure S2 and Supplementary Table S5). Moreover, unlike a *rad55*Δ *dmc1*Δ double mutant, which shows a 10-fold reduction in spore viability, *rlp1*Δ *dmc1*Δ and *rdl1*Δ *dmc1*Δ double mutants display only modest decreases in spore viability compared to wild type (∼2-fold) (Figure 4D and Supplementary Table S5), indicating that DSB repair and meiotic chromosome segregation remain fairly efficient. Altogether these data reveal a more complex interplay between Rad55-Rad57, Rlp1-Rdl1-Sws1 and Swi5-Sfr1 than was suggested by the epistatic relationship between *rad55*Δ and *rlp1*Δ.

### Absence of Rad51 paralogs/mediators allows both Rqh1 and Fml1 to promote NCO recombinant formation

**Figure 5.**
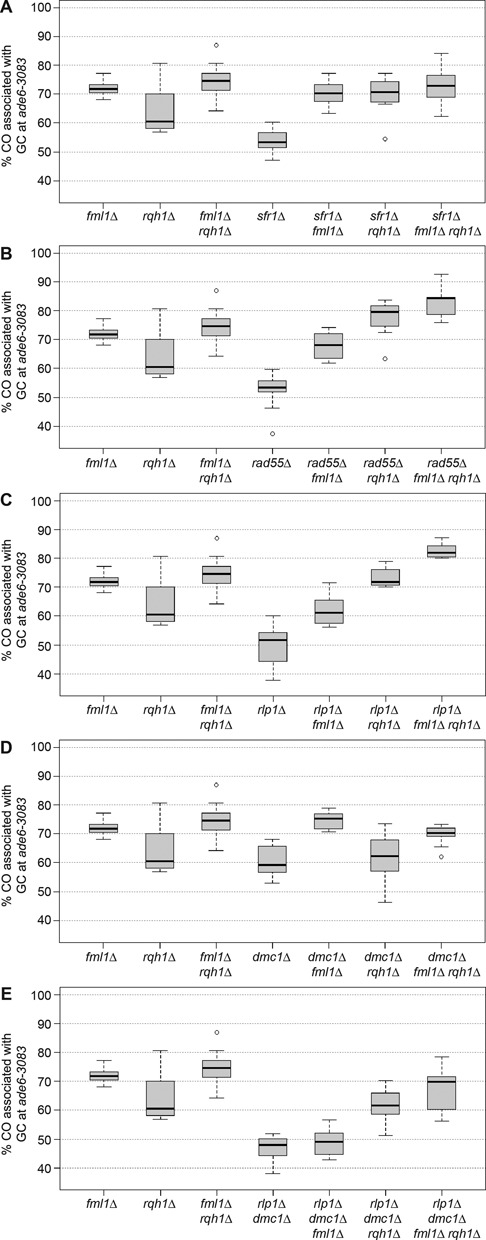
DNA helicases, strand exchange mediators and Dmc1 shape the crossover/non-crossover decision. (**A–E**) Frequency of crossovers associated with a gene conversion event in mutant crosses (*ade6-3083*×*ade6-469*); ALP1133×FO2608 (*fml1*Δ, *n* = 15), ALP781×ALP780 (*rqh1*Δ, *n* = 10), ALP945×ALP944 (*fml1*Δ *rqh1*Δ, *n* = 22). (A) ALP800×ALP782 (*sfr1*Δ, *n* = 22), ALP1134×FO2609 (*sfr1*Δ *fml1*Δ , *n* = 12), ALP801×ALP821 (*sfr1*Δ *rqh1*Δ, *n* = 10), ALP1363×ALP1362 (*sfr1*Δ *fml1*Δ *rqh1*Δ, *n* = 12). (B) ALP1649×ALP1648 (*rad55*Δ, *n* = 11), ALP1658×ALP1657 (*rad55*Δ *fml1*Δ , *n* = 14), UoA328×UoA327 (*rad55*Δ *rqh1*Δ, *n* = 12), UoA330×UoA329 (*rad55*Δ *fml1*Δ *rqh1*Δ, *n* = 9). (C) ALP1623×ALP1620 (*rlp1*Δ, *n* = 18), ALP1664×ALP1663 (*rlp1*Δ *fml1*Δ, *n* = 12), FO3158×FO3159 (*rlp1*Δ *rqh1*Δ, *n* = 10), FO3142×FO3143 (*rlp1*Δ *fml1*Δ *rqh1*Δ, *n* = 10). (D) ALP1545×ALP1544 (*dmc1*Δ, *n* = 10), ALP1590×ALP1589 (*dmc1*Δ *fml1*Δ, *n* = 12), UoA295×UoA294 (*dmc1*Δ *rqh1*Δ, *n* = 8), UoA297×UoA296 (*dmc1*Δ *fml1*Δ *rqh1*Δ, *n* = 11). (E) ALP1694×ALP1693 (*rlp1*Δ *dmc1*Δ, *n* = 12), FO3146×FO3147 (*rlp1*Δ *dmc1*Δ *fml1*Δ, *n* = 10), UoA322×UoA321 (*rlp1*Δ *dmc1*Δ *rqh1*Δ, *n* = 12), UoA299×UoA298 (*rlp1*Δ *dmc1*Δ *fml1*Δ *rqh1*Δ, *n* = 6). *n* indicates the number of independent crosses (see also Supplementary Table S6).

In *sfr1*Δ, *rad55*Δ*, rlp1*Δ and *rdl1*Δ single mutants CO-GC events are 50–52%, this is significantly lower than in wild type (Figures 3C and 4C; for *P* values see Supplementary Table S5). In the case of *sfr1*Δ we have previously shown that this reduction is reversed by deletion of *fml1* (16) (Figure 5A). To determine whether Fml1 is also responsible for suppressing CO-GC in the absence of the Rad51 paralogs we measured the percentage of CO-GC in *fml1*Δ *rad55*Δ, *fml1*Δ *rlp1*Δ and *fml1*Δ *rdl1*Δ double mutants. In each case the percentage of CO-GC increases significantly from the Rad51 paralog mutant level (for *P* values see Supplementary Table S6), however only in a *fml1*Δ *rad55*Δ double mutant does it increase to the ∼70% level observed in a *fml1*Δ single mutant, in the *fml1*Δ *rlp1*Δ and *fml1*Δ *rdl1*Δ double mutants the level increases to ∼60%, which is the same as in wild type (Figure 5B and C and Supplementary Figure S3). These data suggest that both Swi5-Sfr1 and Rad55-Rad57 are needed to similar extents to constrain Fml1's NCO-promoting activity and thereby promote CO-GC, whereas Rlp1 and Rdl1 are required to a lesser extent.

**Figure 6.**
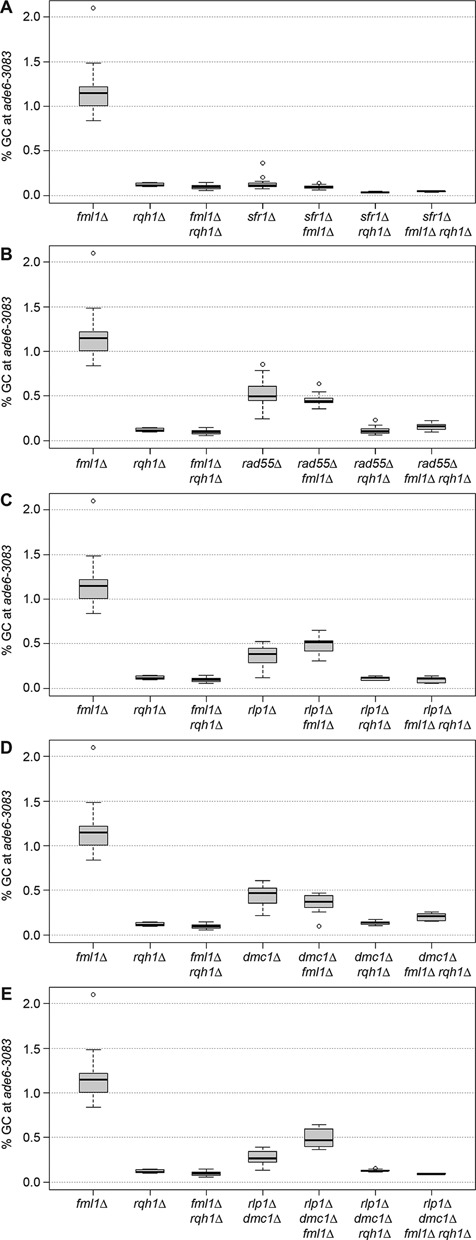
DNA helicases, strand exchange mediators and Dmc1 affect the interhomolog bias. (**A–E**) Percentage of gene conversion in mutant crosses (*ade6-3083*×*ade6-469*), crosses as in Figure 5A–E (see also Supplementary Table S6).

Similar to *rad55*Δ and *rlp1*Δ mutants, a *dmc1*Δ mutant exhibits a moderate decrease in GC and CO frequency (Figure 4A and B and Supplementary Table S5), but unlike them its CO-GC percentage is not reduced (Figure 4C and Supplementary Table S5). This suggests that Dmc1 is not required for constraining Fml1 from promoting NCO formation. Accordingly, the CO-GC percentages in a *dmc1*Δ *fml1*Δ double mutant and *fml1*Δ single mutant are similar (*P* = 0.145) (Figure 5D). However, a different pattern emerges in a *dmc1*Δ *rlp1*Δ double mutant background (Figure 5E). This double mutant exhibits a similar, maybe slightly lower, CO-GC percentage than a *rlp1*Δ mutant, but, unlike in either single mutant, deletion of *fml1* does not increase this percentage to wild-type or higher levels (*P* = 0.687). It does, however, improve GC frequency (*P* = 4.77 × 10^−4^) and cause a synergistic reduction in spore viability (Figure 6E and Supplementary Table S6). The heightened GC frequency of a *dmc1*Δ *rlp1*Δ *fml1*Δ triple compared to a *dmc1*Δ *rlp1*Δ double mutant could indicate that in the absence of both *dmc1* and *rlp1* Fml1 becomes capable of driving template choice away from the homologous chromosome toward the sister chromatid.

RecQ-type helicases in other organisms have been implicated in promoting NCOs during meiosis (19,20,25). However, deletion of the RecQ-type helicase in *S. pombe* Rqh1 has no significant effect on the percentage of CO-GC in either a wild type (*P* = 0.631) or a *fml1*Δ mutant (*P* = 0.458) (Figure 5) (16) even though it strongly limits COs in mitotic cells (56). To see whether Rqh1 might be similarly constrained as Fml1 during meiosis, we measured the percentage of CO-GC in double mutants of *rqh1*Δ with *sfr1*Δ, *rad55*Δ and *rlp1*Δ (Figure 5A–C). In each case CO-GCs increased to 70% or more (for *P* values see Supplementary Table S6). Unlike *fml1*, deletion of *rqh1* in a *dmc1*Δ *rlp1*Δ mutant background also resulted in a significant increase in CO-GC percentage (*P* = 1.63 × 10^−5^) (Figure 5E). The deletion of *fml1* in *rqh1*Δ *rad55*Δ, *rqh1*Δ *rlp1*Δ and *rqh1*Δ *dmc1*Δ *rlp1*Δ mutants resulted in a further increase in CO-GC percentage, whereas no additional change was observed in a *fml1*Δ *rqh1*Δ *sfr1*Δ triple mutant (Figure 5). These data indicate that Rqh1 can promote NCO formation during meiosis, but is normally constrained from doing so by Rad51 paralogs/mediators. Moreover, Rqh1 and Fml1 can seemingly promote NCOs by both common and separate means depending on which Rad51 paralogs/mediators are present.

Consistent with previous findings a *rqh1Δ* mutant exhibits an ∼10-fold reduction in GC (*P* = 1.32 × 10^−11^) and ∼2.5-fold reduction in CO (*P* = 1.62 × 10^−4^) compared with wild type (Figure 6 and Supplementary Table S6) (57). This loss of recombinogenic activity is not associated with a decline in DSB formation or repair, a change in the ratio of interhomolog versus intersister recombination or a dramatic reduction in spore viability (Supplementary Table S6) (57). Instead it has been proposed that Rqh1 might promote hybrid DNA formation necessary for GC (57). This putative function of Rqh1 would appear to operate alongside Rad55 and Rlp1 to support GC formation as both *rqh1*Δ *rad55*Δ (*P* = 0.80) and *rqh1*Δ *rlp1*Δ (*P* = 0.70) double mutants exhibit similar GC frequencies as a *rqh1*Δ single mutant (Figure 6B and C). However, a *rqh1*Δ *rad55*Δ double mutant exhibits a synergistic reduction in spore viability (Supplementary Table S6) indicating that Rqh1 and Rad55 also have non-overlapping roles in promoting reproductive success. A *rqh1*Δ *sfr1*Δ double mutant exhibits a 3- to 4-fold reduction in GC frequency compared to a *rqh1*Δ (*P* = 2.38 × 10^−8^) or a *sfr1*Δ (*P* = 1.97 × 10^−4^) single mutant (Figure 6A). At least in part this can be explained by an additive effect of a weakened interhomolog bias and reduced hybrid DNA formation during interhomolog recombination.

### The Rlp1–Rdl1–Sws1 complex, Dmc1, and Fml1 limit hybrid DNA formation driven in part by Rqh1

**Figure 7.**
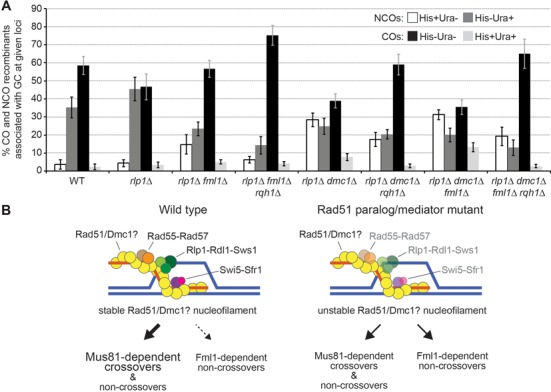
Double and triple mutant combinations of *rlp1*, *dmc1* and *fml1* show a skew in non-crossover recombination classes. (**A**) Frequencies of different crossover and non-crossover classes associated with a gene conversion event in wild-type and mutant crosses (*ade6-3083*×*ade6-469*); ALP733×ALP731 (WT, *n* = 41), ALP1623×ALP1620 (*rlp1*Δ, *n* = 18), ALP1664×ALP1663 (*fml1*Δ *rlp1*Δ, *n* = 12), FO3142×FO3143 (*fml1*Δ *rlp1*Δ *rqh1*Δ, *n* = 10), ALP1694×ALP1693 (*dmc1*Δ *rlp1*Δ, *n* = 12), UoA322×UoA321 (*dmc1*Δ *rlp1*Δ *rqh1*Δ, *n* = 12), FO3146×FO3147 (*dmc1*Δ *fml1*Δ *rlp1*Δ, *n* = 10), UoA299×UoA298 (*dmc1*Δ *fml1*Δ *rlp1*Δ *rqh1*Δ, *n* = 6). *n* indicates the number of independent crosses (see also Supplementary Table S7). (**B**) Model depicting possible outcomes of processing a D-loop in the presence or absence of Rad51 paralogs/mediators. Whether strand exchange by both Rad51- and Dmc1-nucleofilaments is supported by Rad55-57 and/or Rlp1-Rdl1-Sws1 has not been characterized biochemically yet. Thickness and style of arrows indicates the prevalence of Mus81-dependent cleavage or Fml1-dependent non-crossover formation via synthesis-dependent strand annealing (SDSA). DNA strands are shown as blue and orange lines.

Not only are the overall levels of CO-GC events strongly decreased in *dmc1*Δ *rlp1*Δ double mutants (Figure 5E), but the distribution of NCO recombinants among GCs is also starkly different from that in wild-type or either single mutant (Figure 7A). In wild type, the vast majority of NCO-Ade^+^ recombinants are linked to the flanking marker of the hotspot allele (i.e. *ura4^+^-aim2*). This can be explained by the formation of hybrid DNA not extending as far as the cold allele (i.e. *ade6-469*), as envisaged in Supplementary Figure S1 (scenarios 1–4 and 6–9). However, in *dmc1*Δ *rlp1*Δ and *dmc1*Δ *rdl1*Δ double mutants, and to some extent in *fml1*Δ *rlp1*Δ and *fml1*Δ *rdl1*Δ double mutants, a high proportion of NCO-GC recombinants are linked to the flanking marker of the cold allele (Figure 7A and Supplementary Figure S4), this skew is even more pronounced in a *dmc1*Δ *rlp1*Δ *fml1*Δ triple mutant (Figure 7A). One likely explanation for this is extensive branch migration beyond the position of the cold allele (Supplementary Figure S1, scenarios 5 and 10). These data suggest that the Rlp1–Rdl1(–Sws1) complex and Dmc1 perform redundant roles in limiting hybrid DNA formation, with an additional contribution by Fml1. Deletion of *rqh1* in *dmc1*Δ *rlp1*Δ and *dmc1*Δ *rlp1*Δ *fml1*Δ backgrounds, while significantly reducing the overall frequency of NCO-GCs, does not restore their wild-type ratio (Figure 7A). These data are consistent with Rqh1's proposed role in promoting hybrid DNA formation, but also suggest that extensive hybrid DNA can be formed without it when Dmc1 and Rlp1 are absent.

## DISCUSSION

### The Rad51 paralogs/mediators in meiotic recombination

In agreement with previous studies (52,53) we have shown that Rad55-Rad57 and Swi5-Sfr1 have both distinct and overlapping/compensatory functions for promoting meiotic recombination. Both protein complexes are capable of mediating the nucleation of Rad51 onto RPA-coated ssDNA and enhancing nucleoprotein filament stability, albeit Swi5-Sfr1 is a much better mediator for Dmc1 than it is for Rad51 (35,36,58). Accordingly, *sfr1* and *dmc1* exhibit an epistatic interaction for meiotic recombination and spore viability, as opposed to the synergistic interaction observed between *rad55* and *dmc1*. Epistasis analysis also indicates that the Rlp1–Rdl1–Sws1 complex (42) functions together with Rad55-Rad57, which accords with recent data on the homologous complex in budding yeast (comprised of Shu1, Shu2, Psy3 and Csm2), which was shown to physically interact with Rad55-Rad57 and function epistatically with it for mitotic recombination (59). Similar to Rad55-Rad57, Psy3-Csm2 enhance Rad51 nucleoprotein filament stability *in vitro* and plays an important role in promoting meiotic recombination, which becomes essential in the absence of *dmc1* (37,54). However, unlike its budding yeast equivalent and in contrast to a *rad55*Δ *dmc1*Δ double mutant, a *rlp1*Δ *dmc1*Δ double mutant in fission yeast still exhibits moderate levels of meiotic recombination and spore viability. One possible explanation is that in fission yeast the Rlp1–Rdl1–Sws1 complex imposes some kind of constraint over Swi5-Sfr1 assisting Rad51 nucleoprotein filament formation and function. It is also possible that the absence of Dmc1 allows Swi5-Sfr1 to support Rad51-mediated recombination more often. Swi5-Sfr1 loads and stabilizes Dmc1 filaments, whereas it only stabilizes, but does not load, Rad51 filaments (35), and consequently Swi5-Sfr1 might tend to promote Dmc1- rather than Rad51-driven recombination because of its earlier association with Dmc1. If these constraints are removed Swi5-Sfr1 might be available to promote Rad51 activity (either together with or in parallel to Rad55-Rad57) sufficiently well to partially compensate for the lack of Rlp1 and Dmc1. This potential interplay between Rad51 paralogs and mediators, influencing the contributions that Rad51 and Dmc1 make to meiotic recombination, contrasts with the situation in *S. cerevisiae* where Rad51 itself functions as a mediator of Dmc1-driven strand exchange (60), and its own strand exchange activity is massively curbed to avoid competition between it and Dmc1. This is achieved by a specialized and *Saccharomyces*-specific factor, called Hed1, which downregulates Rad51's activity (61–63).

Based on our data deletion of any of the Rad51/Dmc1 paralogs causes a significant reduction in intragenic recombination. This contradicts a previous study, which found no effect of deleting *rad55*/*rad57* or *rlp1* on GC frequency (64). We suspect that this difference is due to analyzing GC at a hotspot allele (our study) versus a non-hotspot allele (64), and could relate to the phenomenon of CO invariance (53). According to CO invariance, recombination at non-hotspots, but not at hotspots, strongly depends on Dmc1 (53). Together with the observation that Rad55-Rad57 and Rlp1 play a less important role for GC formation at non-hotspots (64), one might surmise that there is a division of labor between Dmc1 and Rad55-Rad57 plus Rdl1-Rlp1-Sws1 at sites with different levels of meiotic recombination competence. However, our data indicate that these suggested relationships do not hold true at the *ade6-3083* hotspot. In wild type a similar level of GC frequency is observed at the *ade6-3083* hotspot compared with the *ade6-3049* hotspot (∼1.3% versus ∼1.4%), whereas in a *dmc1*Δ mutant GC rate is reduced 3-fold (*P* = 2.88 × 10^−8^) at *ade6-3083* (Figure 4A and Supplementary Table S5) in contrast to *ade6-3049* where it is reduced by only 1.2-fold (53). Intriguingly, the Smith lab reported a 3-fold reduction in GC frequency for the coldest *ade6* allele combination (*3057* × *M375*) they tested in a wild-type versus *dmc1*Δ comparison (53), similar to what we see at the *ade6-3083* hotspot.

### Swi5-Sfr1 and Rad51 paralogs function together to restrict Fml1 promoting NCO formation

We have previously shown that Fml1 restricts CO-GC percentage in wild-type meiosis most likely by directing repair via SDSA (16). However, its ability to do this is normally restricted by Swi5-Sfr1, and consequently it cannot compensate for any loss of Mus81-Eme1, which results in meiotic catastrophe due to unprocessed recombination intermediates (16,21). The finding that *mus81*Δ spore viability is dramatically improved following the deletion not only of *sfr1* but also *rad55*, *rlp1*, *rdl1* and *dmc1*, without resulting in a recovery of CO formation, suggests that Swi5-Sfr1 and the Rad51 paralogs function together to constrain Fml1 and possibly other DNA helicases from processing recombination intermediates (Figure 7B). Our favored model is that they physically exclude Fml1 from accessing D-loop DNA by promoting the formation and stabilization of Rad51- (and Dmc1-) nucleoprotein filaments. Consistent with this model deletion of *sfr1* or most of the Rad51-paralog genes reduces CO-GC percentage in a Fml1-dependent manner. One exception is *dmc1*, which, as already mentioned, is not required for CO-GC formation during meiotic recombination in fission yeast.

### Rqh1 might promote NCOs by driving hybrid DNA formation

Unlike Fml1 loss of Rqh1 does not result in a significant increase in CO-GC percentage in a wild-type background. However, in both *sfr1*Δ and Rad51-paralog mutant backgrounds increases in CO-GC percentage are observed upon removal of *rqh1*. At first glance this could indicate that Rqh1, like Fml1, drives NCO formation but is normally prevented from doing so by fully stabilized Rad51- and Dmc1-nucleoprotein filaments. However, it has been postulated that Rqh1 promotes hybrid DNA formation (57), and one way this could be achieved is through driving the conversion of D-loops into HJs, which could then branch migrate away from the site of strand invasion. In our assay such an activity if left unfettered could lower the CO-GC percentage by decreasing the number of D-loops that are resolved by Mus81-Eme1 and forcing more HJ resolution, which would generate equal numbers of COs and NCOs (Supplementary Figure S1; less of scenarios 2 and 7 and more of scenarios 3–5 and 8–9). The conversion of D-loops into HJs might normally be controlled through the combined action of Swi5-Sfr1 and the Rad51 paralogs, and an impairment of the D-loop stabilization function of these factors might promote a more rapid conversion rate, providing less opportunity for D-loop cleavage. However, some D-loops may remain accessible to Fml1 enabling it to promote NCO formation via the SDSA pathway. The additive increase in CO-GC percentage observed in both *rad55*Δ *fml1*Δ *rqh1*Δ and *rlp1*Δ *fml1*Δ *rqh1*Δ triple mutants compared to the *rad55*Δ *fml1*Δ (*P* = 1.42 × 10^−5^) and *rlp1*Δ *fml1*Δ (*P* = 4.45 × 10^−6^) double mutants, respectively (Figure 5B and C), might therefore be explained by the combined effect of less D-loop unwinding and conversion to HJs, resulting in more D-loops being available for cleavage by Mus81-Eme1 (Supplementary Figure S1—scenarios 2 and 7).

Once formed the branch migration of the HJ would either extend or limit the amount of hybrid DNA that is formed depending on its direction. Rlp1-Rdl1-Sws1 and Dmc1 appear to play overlapping roles in limiting hybrid DNA formation based on the increase in the proportion of His^+^ Ura^−^ GC recombinants observed in *rlp1*Δ/*rdl1*Δ *dmc1*Δ double mutants (Figure 7A, Supplementary Figure S4 and Supplementary Figure S1—scenarios 5 and 10). The rapid conversion of all D-loops into HJs would prevent Fml1 from driving NCO formation via its D-loop unwinding activity, which could explain why no increase in CO-GC percentage is observed in a *rlp1*Δ *dmc1*Δ *fml1*Δ triple mutant. However, Fml1's inability to promote NCOs in the absence of Rlp1 and Dmc1 is seemingly restored upon deletion of Rqh1 based on the additive increase in CO-GC percentage observed in a *rlp1*Δ *dmc1*Δ *rqh1*Δ *fml1*Δ quadruple mutant (Figure 5E). Intriguingly, the presence of *fml1* is required to maintain the reasonably high levels of spore viability in a *rlp1*Δ *dmc1*Δ mutant even though its NCO-promoting activity appears to be inactive (Supplementary Table S6). Possible explanations are that either the efficiency of overall DNA repair is strongly negatively affected in a *rlp1*Δ *dmc1*Δ *fml1*Δ triple mutant leaving unrepaired DSBs or that Fml1 might promote the resolution of HJs by Mus81-Eme1 by catalyzing their branch migration to existing DNA strand discontinuities. Further studies are needed to test the validity of these ideas.

## CONCLUSION

The role of the Rad51 paralogs/mediators in promoting homologous recombination is well established; however their role in directing homologous recombination outcome by influencing the processing of recombination intermediates is only recently becoming recognized. A common theme that is emerging is one where Rad51 paralogs/mediators play a role in promoting Rad51-nucleoprotein filament stability, which restricts the ability of DNA helicases/translocases to act. This has been observed *in vitro* for human RAD51B and RAD51C limiting the antirecombinogenic activity of the RecQ-type helicase BLM on RAD51 (65), and for budding yeast Rad55-Rad57 enabling Rad51 to resist displacement from DNA by the Srs2 DNA helicase (36). Similarly Rad51 paralogs/mediators appear to antagonize the Rad51 nucleoprotein filament disrupting activity of the Fbh1 DNA helicase in fission yeast (66,67). While these studies reveal the important role that Rad51 paralogs play in preventing DNA helicases from aborting homologous recombination, our study highlights how their role in promoting Rad51-nucleoprotein filament stability may also be important for controlling D-loop processing. We propose that the full ensemble of Rad51 paralogs together with Swi5-Sfr1 are required to create and maintain a Rad51(/Dmc1?)-nucleoprotein filament that remains permissive to Mus81-Eme1 nuclease activity but at the same time acts to occlude and/or constrain Fml1 and Rqh1 activity on the underlying D-loop. Modulation of the recruitment of these proteins during Rad51(/Dmc1?)-nucleoprotein filament assembly could therefore provide a mechanism for influencing both hybrid DNA formation and the CO/NCO decision.

## AVAILABILITY

Supporting online material for this publication is available at http://dx.doi.org/10.6084/m9.figshare.1197171.

## SUPPLEMENTARY DATA

Supplementary Data are available at NAR Online.

SUPPLEMENTARY DATA
