# Supplementary material for: Rad51/Dmc1 paralogs and mediators oppose DNA helicases to limit hybrid DNA formation and promote crossovers during meiotic recombination
Source: Nucleic Acids Res. 2014 Nov 20;42(22):13723–35. doi: 10.1093/nar/gku1219 (PMC4267644; doi:10.1093/nar/gku1219)
Supplement: SUPPLEMENTARY DATA [file supp_42_22_13723__index.html]

Rad51/Dmc1 paralogs and mediators oppose DNA helicases to limit hybrid DNA formation and promote crossovers during meiotic recombination — Rad51/Dmc1 paralogs and mediators oppose DNA helicases to limit hybrid DNA formation and promote crossovers during meiotic recombination — SUPPLEMENTARY DATA 

# Rad51/Dmc1 paralogs and mediators oppose DNA helicases to limit hybrid DNA formation and promote crossovers during meiotic recombination

## SUPPLEMENTARY DATA

**Files in this Data Supplement:**

- SUPPLEMENTARY DATA
- SUPPLEMENTARY DATA
- SUPPLEMENTARY DATA
