# Supplementary material for: Rad51/Dmc1 paralogs and mediators oppose DNA helicases to limit hybrid DNA formation and promote crossovers during meiotic recombination
Source: Nucleic Acids Res. 2014 Nov 20;42(22):13723–35. doi: 10.1093/nar/gku1219 (PMC4267644; doi:10.1093/nar/gku1219)
Supplement: SUPPLEMENTARY DATA [file supp_gku1219_nar-02626-v-2014-File009.pdf]

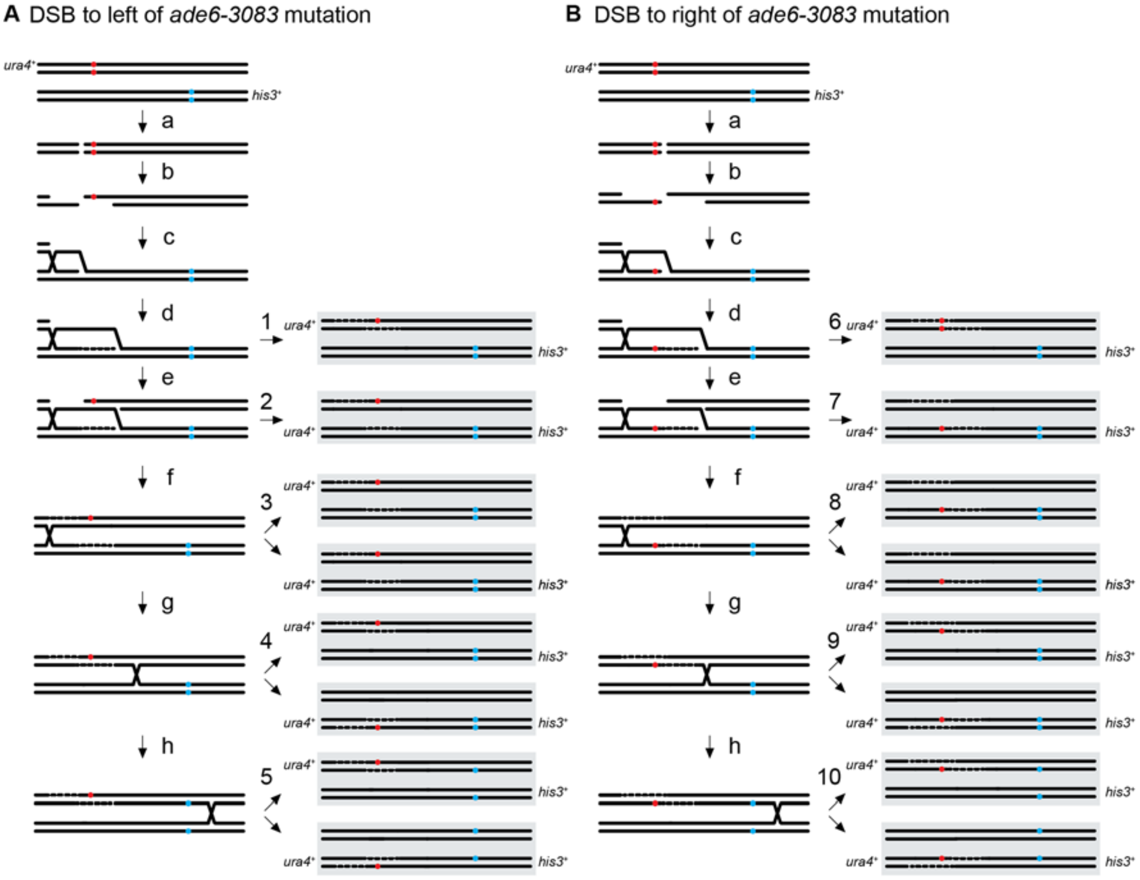

Key

a: DSB formation

b: DSB resection

c: Strand invasion forming a D-loop

d: DNA synthesis primed by the 3' end of the invading strand extends the D-loop

e: Annealing of the other end of the DSB to the displaced strand of the D-loop (second end capture)

f: Processing of the D-loop to form a single HJ

g: Branch migration of the HJ towards the *ade6-469* point mutation

h: Branch migration of the HJ past the *ade6-469* point mutation

C

Scenario	Mechanism	Ade^+ recombinants			
		Ura^+ His^-	Ura^- His^+	Ura^+ His^+	Ura^- His^-
1	SDSA	100%	0	0	0
2	Mus81 D-loop cleavage	0	0	0	100%
3	Mus81 HJ cleavage	50%	0	0	50%
4	Mus81 HJ cleavage	66.6%	0	0	33.3%
5	Mus81 HJ cleavage	16.6%	33.3%	16.6%	33.3%
6	SDSA	0	0	0	0
7	Mus81 D-loop cleavage	0	0	0	100%
8	Mus81 HJ cleavage	50%	0	0	50%
9	Mus81 HJ cleavage	33.3%	0	0	66.6%
10	Mus81 HJ cleavage	16.6%	33.3%	16.6%	33.3%

Figure S1. Possible scenarios for CO/NCO recombination events creating Ade^+ progeny from crosses with different *ade6* heteroalleles and *ura4*$^+$*-aim2* and *his3*$^+$*-aim* as flanking markers

(A, B) The two black lines represent double-stranded DNA of one chromatid; chromatids not involved in the depicted recombination event are omitted for clarity. *ade6-3083* hotspot allele in red, and *ade6-469* non-hotspot allele in light blue. **(C)** Frequency of possible recombination outcomes in crosses involving two *ade6* heteroalleles (*3083* and *469*) and flanking markers (*ura4*$^+$*-aim2* and *his3*$^+$*-aim*) as shown in (A) and (B) assuming mismatch repair-directed correction of point mutations in heteroduplex DNA is equally probable in either direction.

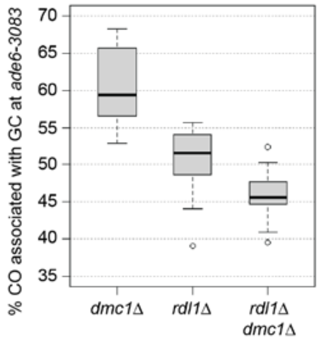

Figure S2. ***rlp1*** **and** ***rdl1*** **show a similar genetic interaction with** ***dmc1***
Frequency of crossovers associated with a gene conversion event in mutant crosses (*ade6-3083*×*ade6-469*); ALP1545×ALP1544 (*dmc1*Δ, n = 12), ALP1621×ALP1611 (*rdl1*Δ, n = 18), ALP1692×ALP1691 (*dmc1*Δ *rdl1*Δ, n = 12). n indicates the number of independent crosses (see also Supplementary Table S5).

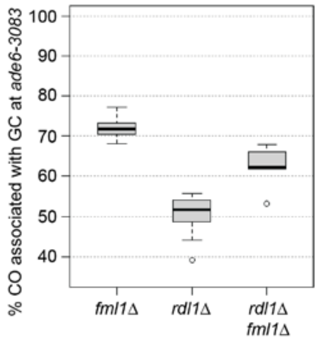

Figure S3. ***rlp1*** **and** ***rdl1*** **show a similar genetic interaction with** ***fml1***
Frequency of crossovers associated with a gene conversion event in mutant crosses (*ade6-3083*×*ade6-469*); ALP1133×FO2608 (*fml1*Δ, n = 15), ALP1621×ALP1611 (*rdl1*Δ, n = 18), ALP1660×ALP1659 (*fml1*Δ *rdl1*Δ, n = 6). n indicates the number of independent crosses (see also Supplementary Table S6).

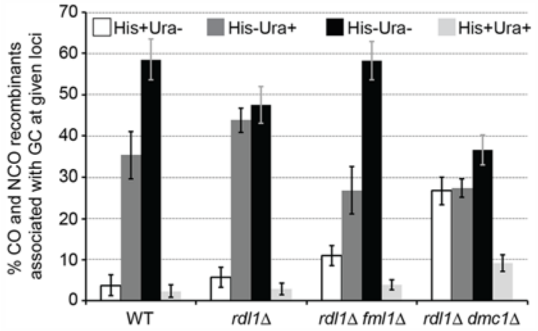

Figure S4. ***rlp1*** **and** ***rdl1*** **show a similar genetic interaction with** ***fml1*** **and** ***dmc1***
Frequencies of different crossover and non-crossover classes associated with a gene conversion event in mutant crosses (*ade6-3083*×*ade6-469*); ALP733×ALP731 (WT, n = 41), ALP621×ALP1611 (*rdl1*Δ, n = 18), ALP1660×ALP1659 (*fml1*Δ *rdl1*Δ, n = 6), ALP1692×ALP1691 (*dmc1*Δ *rdl1*Δ, n = 12). n indicates the number of independent crosses (see also Supplementary Table S7).

Table S1. Yeast strain list (in order of appearance)

Strain	Relevant genotype	Origin
FO652	h^{-smt0} *arg3-D4 his3-D1 leu1-32 ura4-D18*	lab strain
FO656/MCW1221	h^{+N} *arg3-D4 his3-D1 leu1-32 ura4-D18*	lab strain
ALP1593/MCW6065	h^{+S} *ade7-50 arg3-D4 his3-D1 ura4-D18*	this study
ALP1596/MCW6068	h^{-smt0} *ade7-152 his3-D1 leu1-32 ura4-D18*	this study
ALP733/MCW3202	h^{+S} *ade6-3083* $ura4^{+}$*-aim2 his3-D1 leu1-32 ura4-D18*	(1)
ALP731/MCW3200	h^{-smt0} *ade6-469* $his3^{+}$*-aim arg3-D4 his3-D1 ura4-D18*	(1)
ALP1133/MCW4881	h^{+S} *fml1Δ::hphMX4 ade6-3083* $ura4^{+}$*-aim2 his3-D1 leu1-32 ura4-D18*	(2)
FO2608/MCW4718	h^{-smt0} *fml1Δ::hphMX4 ade6-469* $his3^{+}$*-aim arg3-D4 his3-D1 ura4-D18*	(2)
ALP1541/MCW5788	h^{+N} *ade6-M375* $ura4^{+}$*-aim2 his3-D1 leu1-32 ura4-D18*	(2)
ALP1542/MCW5789	h^{+N} *fml1Δ::hphMX4 ade6-M375* $ura4^{+}$*-aim2 his3-D1 leu1-32 ura4-D18*	(2)
ALP1638/MCW6193	h^{+S} $ura4^{+}$*-aim6 ade7-50 arg3-D4 his3-D1 ura4-D18*	this study
ALP1636/MCW6191	h^{-smt0} $his3^{+}$*-aim3 ade7-152 his3-D1 leu1-32 ura4-D18*	this study
ALP1670/MCW6270	h^{+S} *fml1Δ::hphMX4* $ura4^{+}$*-aim6 ade7-50 arg3-D4 his3-D1 ura4-D18*	this study
ALP1669/MCW6269	h^{-smt0} *fml1Δ::hphMX4* $his3^{+}$*-aim3 ade7-152 his3-D1 leu1-32 ura4-D18*	this study
ALP802/MCW3514	h^{+S} *mus81Δ::kanMX6 ade6-3083* $ura4^{+}$*-aim2 his3-D1 leu1-32 ura4-D18*	(1)
ALP822/MCW3589	h^{-smt0} *mus81Δ::kanMX6 ade6-469* $his3^{+}$*-aim arg3-D4 his3-D1 ura4-D18*	(1)
ALP812/MCW3542	h^{-S} *mus81Δ::kanMX6*	(2)
ALP813/MCW3543	h^{-smt0} *mus81Δ::kanMX6*	(2)
ALP824/MCW3591	h^{+S} *mus81Δ::kanMX6 sfr1Δ-2::natMX4 ade6-3083* $ura4^{+}$*-aim2 his3-D1 leu1-32 ura4-D18*	(2)
ALP823/MCW3590	h^{-smt0} *mus81Δ::kanMX6 sfr1Δ-2::natMX4 ade6-469* $his3^{+}$*-aim arg3-D4 his3-D1 ura4-D18*	(2)
ALP820/MCW3587	h^{-S} *mus81Δ::kanMX6 sfr1Δ-2::natMX4*	(2)
ALP814/MCW3544	h^{-smt0} *mus81Δ::kanMX6 sfr1Δ-2::natMX4*	(2)
ALP1674/MCW6274	h^{+S} *mus81Δ::kanMX6 rad55Δ::hphMX ura4+-aim2 ade6-3083 his3-D1 leu1-32 ura4-D18*	this study[1]
ALP1673/MCW6273	h^{-smt0} *mus81Δ::kanMX6 rad55Δ::hphMX his3+-aim ade6-469 arg3-D4 his3-D1 ura4-D18*	this study[1]
ALP1676/MCW6276	h^{+S} *mus81Δ::kanMX6 rdl1Δ-25::natMX6 ade6-3083* $ura4^{+}$*-aim2 his3-D1 leu1-32 ura4-D18*	this study
ALP1675/MCW6275	h^{-smt0} *mus81Δ::kanMX6 rdl1Δ-25::natMX6 ade6-469* $his3^{+}$*-aim arg3-D4 his3-D1 ura4-D18*	this study
ALP1680/MCW6280	h^{+S} *mus81Δ::kanMX6 rlp1Δ-7::natMX6 ade6-3083* $ura4^{+}$*-aim2 his3-D1 leu1-32 ura4-D18*	this study
ALP1679/MCW6279	h^{-smt0} *mus81Δ::kanMX6 rlp1Δ-7::natMX6 ade6-469* $his3^{+}$*-aim arg3-D4 his3-D1 ura4-D18*	this study
ALP1672/MCW6272	h^{+S} *dmc1Δ-12::natMX4 mus81Δ::kanMX6 ade6-3083* $ura4^{+}$*-aim2 his3-D1 leu1-32 ura4-D18*	this study
ALP1671/MCW6271	h^{-smt0} *dmc1Δ-12::natMX4 mus81Δ::kanMX6 ade6-469* $his3^{+}$*-aim arg3-D4 his3-D1 ura4-D18*	this study
ALP1623/MCW6178	h^{+S} *rlp1Δ-7::natMX6 ura4+-aim2 ade6-3083 his3-D1 leu1-32 ura4-D18*	this study
ALP1620/MCW6169	h^{-smt0} *rlp1Δ-7::natMX6 his3+-aim ade6-469 arg3-D4 his3-D1 ura4-D18*	this study
ALP1621/MCW6170	h^{+S} *rdl1Δ-25::natMX6 ura4+-aim2 ade6-3083 his3-D1 leu1-32 ura4-D18*	this study
ALP1611/MCW6155	h^{-smt0} *rdl1Δ-25::natMX6 his3+-aim ade6-469 arg3-D4 his3-D1 ura4-D18*	this study
ALP1708/MCW6369	h^{+S} *sws1Δ-23::kanMX6 ura4+-aim2 ade6-3083 his3-D1 leu1-32 ura4-D18*	this study
ALP1707/MCW6368	h^{-smt0} *sws1Δ-23::kanMX6 his3+-aim ade6-469 arg3-D4 his3-D1 ura4-D18*	this study
ALP1690/MCW6323	h^{+S} *rdl1Δ::kanMX6 rlp1Δ-7::natMX6 ura4+-aim2 ade6-3083 his3-D1 leu1-32 ura4-D18*	this study
ALP1689/MCW6322	h^{-smt0} *rdl1Δ::kanMX6 rlp1Δ-7::natMX6 his3+-aim ade6-469 arg3-D4 his3-D1 ura4-D18*	this study
ALP1733/MCW6529	h^{+S} *rdl1Δ::kanMX6 rlp1Δ-7::natMX6 sws1Δ-18::hphMX4 ura4+-aim2 ade6-3083 his3-D1 leu1-32 ura4-D18*	this study
ALP1732/MCW6528	h^{-smt0} *rdl1Δ::kanMX6 rlp1Δ-7::natMX6 sws1Δ-18::hphMX4 his3+-aim ade6-469 arg3-D4 his3-D1 ura4-D18*	this study
ALP800/MCW3500	h^{+S} *sfr1Δ-2::natMX4 ade6-3083* $ura4^{+}$*-aim2 his3-D1 leu1-32 ura4-D18*	(2)
ALP782/MCW3386	h^{-smt0} *sfr1Δ-2::natMX4 ade6-469* $his3^{+}$*-aim arg3-D4 his3-D1 ura4-D18*	(2)
ALP1649/MCW6218	h^{+S} *rad55Δ::hphMX ura4+-aim2 ade6-3083 his3-D1 leu1-32 ura4-D18*	this study[1]
ALP1648/MCW6217	h^{-smt0} *rad55Δ::hphMX his3+-aim ade6-469 arg3-D4 his3-D1 ura4-D18*	this study[1]
ALP1545/MCW5795	h^{+S} *dmc1Δ-12::natMX4 ade6-3083* $ura4^{+}$*-aim2 his3-D1 leu1-32 ura4-D18*	(2)
ALP1544/MCW5793	h^{-smt0} *dmc1Δ-12::natMX4 ade6-469* $his3^{+}$*-aim arg3-D4 his3-D1 ura4-D18*	(2)
ALP1735/MCW6547	h^{+S} *rad55Δ::hphMX sfr1Δ-2::natMX4 ura4+-aim2 ade6-3083 his3-D1 leu1-32 ura4-D18*	this study[1]
ALP1734/MCW6546	h^{-smt0} *rad55Δ::hphMX sfr1Δ-2::natMX4 his3+-aim ade6-469 arg3-D4 his3-D1 ura4-D18*	this study[1]
ALP1700/MCW6333	h^{+S} *rlp1Δ-7::natMX6 sfr1Δ-11::hphMX4 ura4+-aim2 ade6-3083 his3-D1 leu1-32 ura4-D18*	this study
ALP1699/MCW6332	h^{-smt0} *rlp1Δ-7::natMX6 sfr1Δ-11::hphMX4 his3+-aim ade6-469 arg3-D4 his3-D1 ura4-D18*	this study
ALP1588/MCW6053	h^{+S} *dmc1Δ-12::natMX4 sfr1Δ-11::hphMX4 ura4+-aim2 ade6-3083 his3-D1 leu1-32 ura4-D18*	this study
ALP1587/MCW6052	h^{-smt0} *dmc1Δ-12::natMX4 sfr1Δ-11::hphMX4 his3+-aim ade6-469 arg3-D4 his3-D1 ura4-D18*	this study
ALP1704/MCW6337	h^{+S} *rad55Δ::hphMX rlp1Δ-7::natMX6 ura4+-aim2 ade6-3083 his3-D1 leu1-32 ura4-D18*	this study[1]
ALP1703/MCW6336	h^{-smt0} *rad55Δ::hphMX rlp1Δ-7::natMX6 his3+-aim ade6-469 arg3-D4 his3-D1 ura4-D18*	this study[1]
ALP1696/MCW6329	h^{+S} *dmc1Δ-12::natMX4 rad55Δ::hphMX ura4+-aim2 ade6-3083 his3-D1 leu1-32 ura4-D18*	this study[1]
ALP1695/MCW6328	h^{-smt0} *dmc1Δ-12::natMX4 rad55Δ::hphMX his3+-aim ade6-469 arg3-D4 his3-D1 ura4-D18*	this study[1]
ALP1694/MCW6327	h^{+S} *dmc1Δ-12::natMX4 rlp1Δ::kanMX6 ura4+-aim2 ade6-3083 his3-D1 leu1-32 ura4-D18*	this study
ALP1693/MCW6326	h^{-smt0} *dmc1Δ-12::natMX4 rlp1Δ::kanMX6 his3+-aim ade6-469 arg3-D4 his3-D1 ura4-D18*	this study
ALP781/MCW3385	h^{+S} *rqh1Δ::kanMX6 ade6-3083* $ura4^{+}$*-aim2 his3-D1 leu1-32 ura4-D18*	this study
ALP780/MCW3384	h^{-smt0} *rqh1Δ::kanMX6 ade6-469* $his3^{+}$*-aim arg3-D4 his3-D1 ura4-D18*	this study
ALP783/MCW3387	h^{+S} *rqh1Δ::kanMX6*	(2)
ALP784/MCW3388	h^{-smt0} *rqh1Δ::kanMX6*	(2)
ALP945/MCW4176	h^{+S} *fml1Δ::natMX4 rqh1Δ::kanMX6 ade6-3083* $ura4^{+}$*-aim2 his3-D1 leu1-32 ura4-D18*	this study
ALP944/MCW4175	h^{-smt0} *fml1Δ::natMX4 rqh1Δ::kanMX6 ade6-469* $his3^{+}$*-aim arg3-D4 his3-D1 ura4-D18*	this study

ALP991/MCW4477	h^{+S} fml1Δ::natMX4 rqh1Δ::kanMX6	this study
ALP992/MCW4478	h^{-smt0} fml1Δ::natMX4 rqh1Δ::kanMX6	this study
ALP1134/MCW4882	h^{+S} fml1Δ::hphMX4 sfr1Δ-2::natMX4 ade6-3083 $ura4^{+}$-aim2 his3-D1 leu1-32 ura4-D18	(2)
FO2609/MCW4719	h^{-smt0} fml1Δ::hphMX4 sfr1Δ-2::natMX4 ade6-469 $his3^{+}$-aim arg3-D4 his3-D1 ura4-D18	(2)
ALP1135/MCW4885	h^{+S} fml1Δ::hphMX4 sfr1Δ-2::natMX4	(2)
ALP1136/MCW4886	h^{-smt0} fml1Δ::hphMX4 sfr1Δ-2::natMX4	(2)
ALP801/MCW3501	h^{+S} rqh1Δ::kanMX6 sfr1Δ-2::natMX4 ade6-3083 $ura4^{+}$-aim2 his3-D1 leu1-32 ura4-D18	this study
ALP821/MCW3588	h^{-smt0} rqh1Δ::kanMX6 sfr1Δ-2::natMX4 ade6-469 $his3^{+}$-aim arg3-D4 his3-D1 ura4-D18	this study
ALP798/MCW3498	h^{+S} rqh1Δ::kanMX6 sfr1Δ-2::natMX4	this study
ALP799/MCW3499	h^{-smt0} rqh1Δ::kanMX6 sfr1Δ-2::natMX4	this study
ALP1363/MCW5328	h^{+S} fml1Δ::hphMX4 rqh1Δ::kanMX6 sfr1Δ-2::natMX4 ade6-3083 $ura4^{+}$-aim2 his3-D1 leu1-32 ura4-D18	this study
ALP1362/MCW5327	h^{-smt0} fml1Δ::hphMX4 rqh1Δ::kanMX6 sfr1Δ-2::natMX4 ade6-469 $his3^{+}$-aim arg3-D4 his3-D1 ura4-D18	this study
ALP1658/MCW6256	h^{+S} fml1Δ::natMX4 rad55Δ::hphMX ura4+-aim2 ade6-3083 his3-D1 leu1-32 ura4-D18	this study[1]
ALP1657/MCW6255	h^{-smt0} fml1Δ::natMX4 rad55Δ::hphMX his3+-aim ade6-469 arg3-D4 his3-D1 ura4-D18	this study[1]
UoA328	h^{+S} rad55Δ::hphMX rqh1Δ::kanMX6 ade6-3083 $ura4^{+}$-aim2 his3-D1 leu1-32 ura4-D18	this study[1]
UoA327	h^{-smt0} rad55Δ::hphMX rqh1Δ::kanMX6 ade6-469 $his3^{+}$-aim arg3-D4 his3-D1 ura4-D18	this study[1]
UoA330	h^{+S} fml1Δ::natMX4 rad55Δ::hphMX rqh1Δ::kanMX6 ade6-3083 $ura4^{+}$-aim2 his3-D1 leu1-32 ura4-D18	this study[1]
UoA329	h^{-smt0} fml1Δ::natMX4 rad55Δ::hphMX rqh1Δ::kanMX6 ade6-469 $his3^{+}$-aim arg3-D4 his3-D1 ura4-D18	this study[1]
ALP1664/MCW6262	h^{+S} fml1Δ::hphMX4 rlp1Δ-7::natMX6 ura4+-aim2 ade6-3083 his3-D1 leu1-32 ura4-D18	this study
ALP1663/MCW6261	h^{-smt0} fml1Δ::hphMX4 rlp1Δ-7::natMX6 his3+-aim ade6-469 arg3-D4 his3-D1 ura4-D18	this study
FO3158/MCW6595	h^{+S} rlp1Δ-7::natMX6 rqh1Δ::kanMX6 ade6-3083 $ura4^{+}$-aim2 his3-D1 leu1-32 ura4-D18	this study
FO3159/MCW6596	h^{-smt0} rlp1Δ-7::natMX6 rqh1Δ::kanMX6 ade6-469 $his3^{+}$-aim arg3-D4 his3-D1 ura4-D18	this study
FO3142/MCW6579	h^{+S} fml1Δ::hphMX4 rlp1Δ-7::natMX6 rqh1Δ::kanMX6 ade6-3083 $ura4^{+}$-aim2 his3-D1 leu1-32 ura4-D18	this study
FO3143/MCW6580	h^{-smt0} fml1Δ::hphMX4 rlp1Δ-7::natMX6 rqh1Δ::kanMX6 ade6-469 $his3^{+}$-aim arg3-D4 his3-D1 ura4-D18	this study
ALP1590/MCW6055	h^{+S} dmc1Δ-12::natMX4 fml1Δ::hphMX4 ura4+-aim2 ade6-3083 his3-D1 leu1-32 ura4-D18	this study
ALP1589/MCW6054	h^{-smt0} dmc1Δ-12::natMX4 fml1Δ::hphMX4 his3+-aim ade6-469 arg3-D4 his3-D1 ura4-D18	this study
UoA295	h^{+S} dmc1Δ-12::natMX4 rqh1Δ::kanMX6 ade6-3083 $ura4^{+}$-aim2 his3-D1 leu1-32 ura4-D18	this study
UoA294	h^{-smt0} dmc1Δ-12::natMX4 rqh1Δ::kanMX6 ade6-469 $his3^{+}$-aim arg3-D4 his3-D1 ura4-D18	this study
UoA297	h^{+S} dmc1Δ-12::natMX4 fml1Δ::hphMX4 rqh1Δ::kanMX6 ade6-3083 $ura4^{+}$-aim2 his3-D1 leu1-32 ura4-D18	this study
UoA296	h^{-smt0} dmc1Δ-12::natMX4 fml1Δ::hphMX4 rqh1Δ::kanMX6 ade6-469 $his3^{+}$-aim arg3-D4 his3-D1 ura4-D18	this study
FO3146/MCW6583	h^{+S} dmc1Δ-12::natMX4 fml1Δ::hphMX4 rlp1Δ::kanMX6 ura4+-aim2 ade6-3083 his3-D1 leu1-32 ura4-D18	this study
FO3147/MCW6584	h^{-smt0} dmc1Δ-12::natMX4 fml1Δ::hphMX4 rlp1Δ::kanMX6 his3+-aim ade6-469 arg3-D4 his3-D1 ura4-D18	this study
UoA322	h^{+S} dmc1Δ-12::natMX4 rlp1Δ::kanMX6 rqh1Δ::hphMX4 ade6-3083 $ura4^{+}$-aim2 his3-D1 leu1-32 ura4-D18	this study
UoA321	h^{-smt0} dmc1Δ-12::natMX4 rlp1Δ::kanMX6 rqh1Δ::hphMX4 ade6-469 $his3^{+}$-aim arg3-D4 his3-D1 ura4-D18	this study
UoA299	h^{+S} dmc1Δ-12::natMX4 fml1Δ::hphMX4 rlp1Δ-7::natMX6 rqh1Δ::kanMX6 ade6-3083 $ura4^{+}$-aim2 his3-D1 leu1-32 ura4-D18	this study
UoA298	h^{-smt0} dmc1Δ-12::natMX4 fml1Δ::hphMX4 rlp1Δ-7::natMX6 rqh1Δ::kanMX6 ade6-469 $his3^{+}$-aim arg3-D4 his3-D1 ura4-D18	this study
ALP1692/MCW6325	h^{+S} dmc1Δ-12::natMX4 rdl1Δ::kanMX6 ura4+-aim2 ade6-3083 his3-D1 leu1-32 ura4-D18	this study
ALP1691/MCW6324	h^{-smt0} dmc1Δ-12::natMX4 rdl1Δ::kanMX6 his3+-aim ade6-469 arg3-D4 his3-D1 ura4-D18	this study
ALP1660/MCW6258	h^{+S} fml1Δ::hphMX4 rdl1Δ-25::natMX6 ura4+-aim2 ade6-3083 his3-D1 leu1-32 ura4-D18	this study
ALP1659/MCW6257	h^{-smt0} fml1Δ::hphMX4 rdl1Δ-25::natMX6 his3+-aim ade6-469 arg3-D4 his3-D1 ura4-D18	this study

[1]*rad55Δ::hphMX* strains are derivatives of FY18530 provided by the National BioResource Project (NBRP) of the MEXT, Japan.

1. Lorenz, A., West, S.C. and Whitby, M.C. (2010) The human Holliday junction resolvase GEN1 rescues the meiotic phenotype of a Schizosaccharomyces pombe mus81 mutant. *Nucleic Acids Res.*, **38**, 1866–73.

2. Lorenz, A., Osman, F., Sun, W., Nandi, S., Steinacher, R. and Whitby, M.C. (2012) The fission yeast FANCM ortholog directs non-crossover recombination during meiosis. *Science*, **336**, 1585–8.
