# Supplementary material for: Rad51/Dmc1 paralogs and mediators oppose DNA helicases to limit hybrid DNA formation and promote crossovers during meiotic recombination
Source: Nucleic Acids Res. 2014 Nov 20;42(22):13723–35. doi: 10.1093/nar/gku1219 (PMC4267644; doi:10.1093/nar/gku1219)
Supplement: SUPPLEMENTARY DATA [file supp_gku1219_Lorenz2014_Suppl_Figs_S1-4.docx]

**
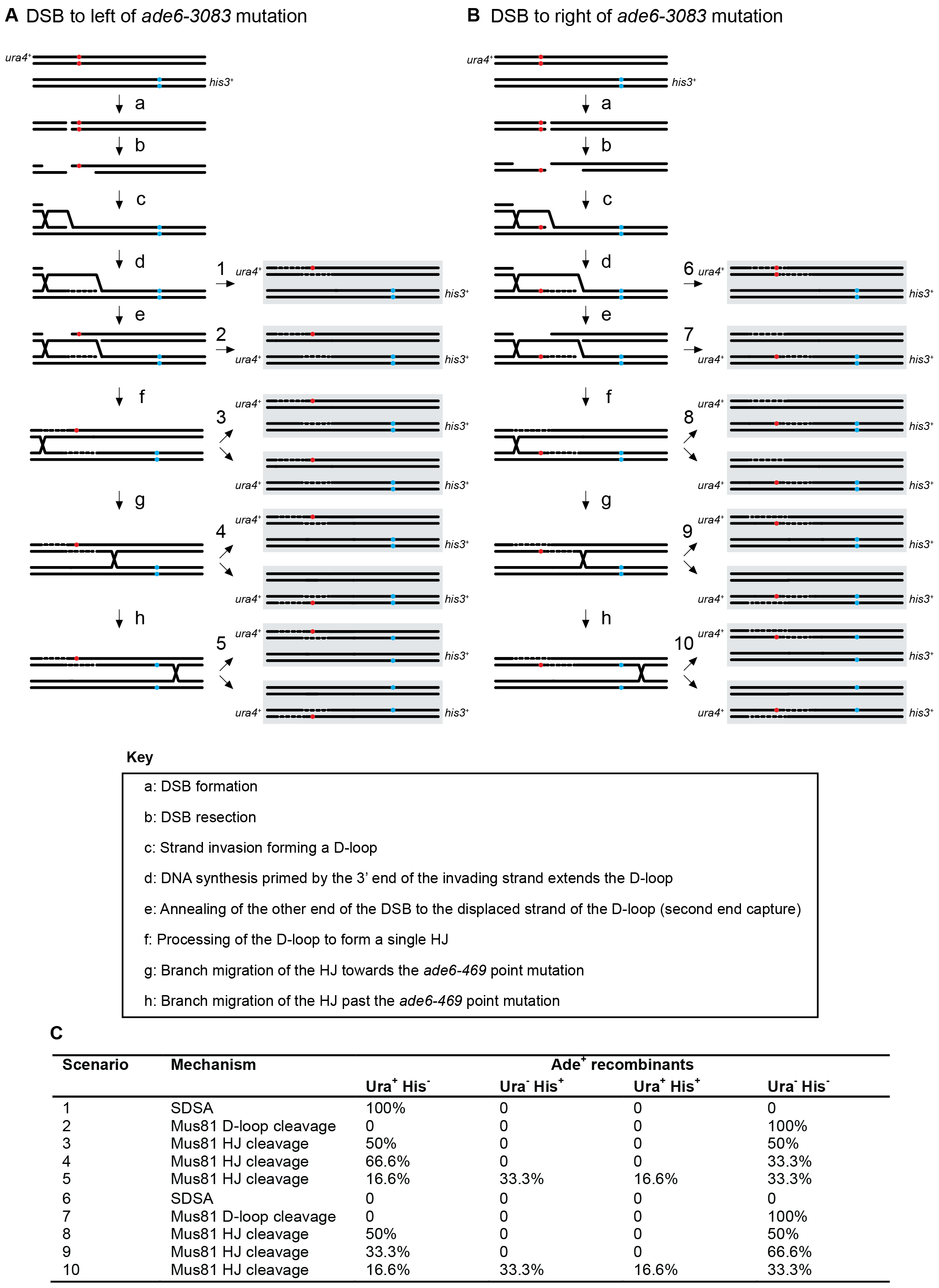
**

**Figure S1. Possible scenarios for CO/NCO recombination events creating Ade^+^ progeny from crosses with different *ade6* heteroalleles and *ura4^+^-aim2* and *his3^+^-aim* as flanking markers**

**(A, B)** The two black lines represent double-stranded DNA of one chromatid; chromatids not involved in the depicted recombination event are omitted for clarity. *ade6-3083* hotspot allele in red, and *ade6-469* non-hotspot allele in light blue. **(C)** Frequency of possible recombination outcomes in crosses involving two *ade6* heteroalleles (*3083* and *469*) and flanking markers (*ura4*^+^*-aim2* and *his3*^+^*-aim*) as shown in (A) and (B) assuming mismatch repair-directed correction of point mutations in heteroduplex DNA is equally probable in either direction.

**
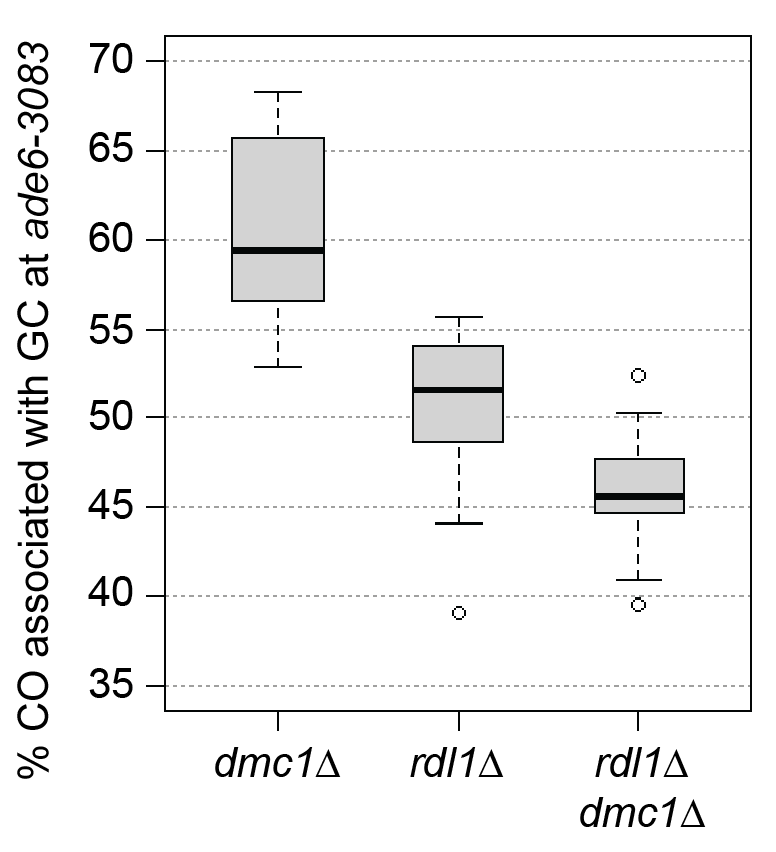
**

**Figure S2. *rlp1* and *rdl1* show a similar genetic interaction with *dmc1***

Frequency of crossovers associated with a gene conversion event in mutant crosses (*ade6-3083*×*ade6-469*); ALP1545×ALP1544 (*dmc1*Δ, n = 12), ALP1621×ALP1611 (*rdl1*Δ, n = 18), ALP1692×ALP1691 (*dmc1*Δ *rdl1*Δ, n = 12). n indicates the number of independent crosses (see also Supplementary Table S5).

**
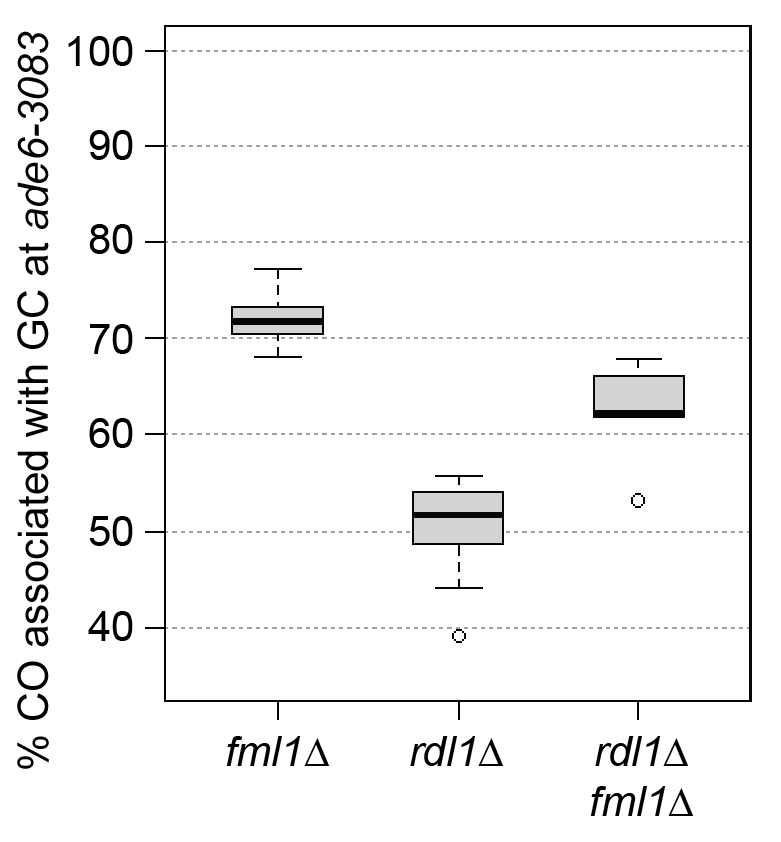
**

**Figure S3. *rlp1* and *rdl1* show a similar genetic interaction with *fml1***

Frequency of crossovers associated with a gene conversion event in mutant crosses (*ade6-3083*×*ade6-469*); ALP1133×FO2608 (*fml1*Δ, n = 15), ALP1621×ALP1611 (*rdl1*Δ, n = 18), ALP1660×ALP1659 (*fml1*Δ *rdl1*Δ, n = 6). n indicates the number of independent crosses (see also Supplementary Table S6).

**
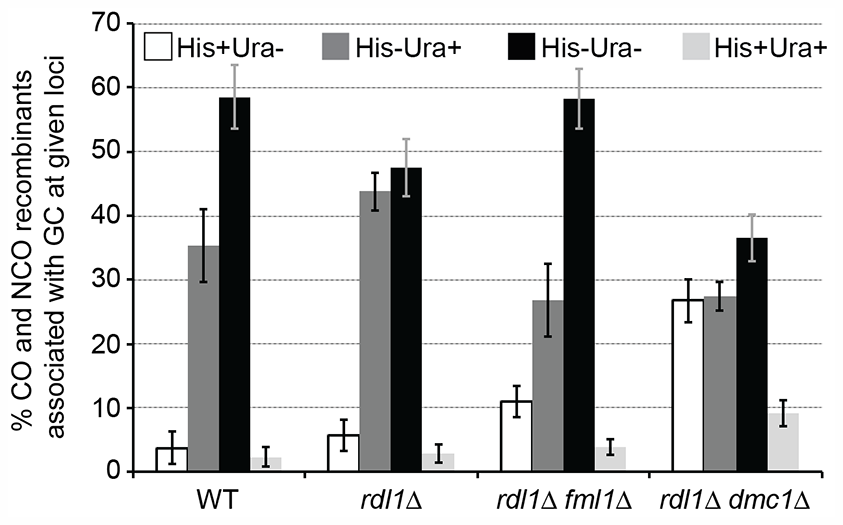
**

**Figure S4. *rlp1* and *rdl1* show a similar genetic interaction with *fml1* and *dmc1***

Frequencies of different crossover and non-crossover classes associated with a gene conversion event in mutant crosses (*ade6-3083*×*ade6-469*); ALP733×ALP731 (WT, n = 41), ALP621×ALP1611 (*rdl1*Δ, n = 18), ALP1660×ALP1659 (*fml1*Δ *rdl1*Δ, n = 6), ALP1692×ALP1691 (*dmc1*Δ *rdl1*Δ, n = 12). n indicates the number of independent crosses (see also Supplementary Table S7).
